# Cognitive Profile of Autism and Intellectual Disorder in Wechsler’s Scales: Meta-Analysis

**DOI:** 10.3390/ejihpe16010012

**Published:** 2026-01-14

**Authors:** Gustavo Mortari Ferreira, Calliandra Maria de Souza Silva, Alexandre Sampaio Rodrigues Pereira, Larissa Sousa Silva Bonasser, Maria Gabriela do Nascimento Araújo, Marcelly de Oliveira Barros, Roniel Sousa Damasceno, Fauston Negreiros, Izabel Cristina Rodrigues da Silva

**Affiliations:** 1 Postgraduate Program in Health Sciences and Technologies, Faculty of Health Sciences and Technology, University of Brasília (UnB), Brasília 2220-900, DF, Brazil; cdssilva@gmail.com (C.M.d.S.S.); prof.alexandresampaio@gmail.com (A.S.R.P.); 2 Academic Unit of Biotechnology Engineering (UAEB), Center for Sustainable Development of the Semi-Arid Region (CDSA), Sumé Campus, Federal University of Campina Grande, Sumé-Paraiba 58540-000, PB, Brazil; 3 Postgraduate Program in Health Sciences, Faculty of Health Sciences and Technology, University of Brasília (UnB), Brasília 72220-900, DF, Brazil; laribonasser@gmail.com; 4 Postgraduate Program in Clinical Psychology and Culture, Institute of Psychology, University of Brasília (UnB), Brasília 72220-900, DF, Brazil; gabi.sikver@gmail.com (M.G.d.N.A.); barrosmarcelly3@gmail.com (M.d.O.B.); ronielsousa1@gmail.com (R.S.D.); fnegreiros@unb.br (F.N.); 5 Clinical Analysis Laboratory, Molecular Pathology Sector, Pharmacy Department, Faculty of Ceilândia, University of Brasília (UnB), Brasília 72220-900, DF, Brazil

**Keywords:** autismautism spectrum disorder, intellectual development disorder, intellectual disability, meta-analysis, neuropsychological assessment, intelligence, IQ, WAIS, WISC, cognitive profile

## Abstract

Autism spectrum disorder (ASD) and intellectual disability (ID) frequently coexist and share heterogeneous cognitive manifestations, yet their specific performance patterns on Wechsler scales remain poorly systematized. This meta-analysis synthesized data from 31 studies using the WISC-IV, WISC-V, WAIS-III, and WAIS-IV to compare cognitive index profiles in individuals with ASD, ID and ASD+ID. Standardized mean differences (Hedges’ g) were calculated using random-effects models, adopting a normative reference of mean 100 and SD 15. Results showed a distinct profile for ASD, with greater impairments in the Processing Speed Index (PSI) and Working Memory Index (WMI), while the Vocabulary Comprehension Index (VCI), Perceptual/Fluid Reasoning Index (PRI/FRI), and Visual Processing Index (VPI) remained close to normative scores. In contrast, ID and ASD+ID exhibited generalized deficits across all indices, with the lowest scores in Full-Scale IQ (FSIQ) and broad effects above g = −2.5. No significant differences emerged between Wechsler versions or age-based test types. Heterogeneity was high in ASD and ID across outcomes, but negligible in ASD+ID due to reduced k. These findings reinforce that ASD presents a specific cognitive pattern, whereas ID and ASD+ID display diffuse impairment, and that Wechsler scales are consistent across versions for identifying these profiles.

## 1. Introduction

Autism spectrum disorder (ASD) is a neurodevelopmental condition characterized by persistent deficits in cognitive and social functioning, accompanied by repetitive and restricted patterns of interests and behaviors. Intellectual disability (ID), in turn, is defined by significant limitations in intellectual functioning (IQ), as well as impairments in adaptive behavior encompassing the pragmatic, social, and conceptual domains (Crippa et al., 2023). Notably, approximately 26 to 30% of individuals on the autism spectrum present comorbidity with ID or exhibit IQ levels comparable to this group (Bougeard et al., 2021).

Recent studies have demonstrated the existence of multiple ASD subtypes, encompassing distinct groups that may or may not exhibit intellectual impairment and other comorbidities, in addition to presenting genotypic and phenotypic variability (Litman et al., 2025). Moreover, both disorders display heterogeneous clinical profiles and considerable variability in symptomatology, developmental trajectories, and cognitive performance, extending beyond their core diagnostic criteria (Masi et al., 2017).

Given the complexity of these presentations, neuropsychological assessment remains the most appropriate method for delineating the individual’s cognitive strengths and weaknesses. Specifically, in cases involving comorbidities between ASD and ID, the evaluation of intellectual functioning through standardized intelligence testing is particularly relevant (Braconnier & Siper, 2021; Yu et al., 2024).

### 1.1. The Role of Wechsler Scales in ASD and ID Psychometric Evaluation

Wechsler Scales (WS) are the most extensively utilized instruments for assessing intelligence worldwide. The theoretical model proposed by Wechsler conceptualizes intelligence as a composite of cognitive processes that enable individuals to interact effectively with their environment, acquire knowledge, and engage in purposeful, goal-directed behavior. Based on these premises, WS are structured into specific indices that encompass various cognitive domains, which, when integrated, yield a comprehensive profile of an individual’s intellectual functioning. The index currently employed globally includes Verbal Comprehension (VCI), Perceptual Reasoning (PRI), which in the WISC-V edition was replaced by Fluid Reasoning (FRI) and Visual Processing (VPI), Working Memory (WMI), and Processing Speed (PSI). In addition, the scales provide estimates of general intellectual ability, such as the Full-Scale Intelligence Quotient (FSIQ), Verbal IQ, and Performance IQ (Barnard, 2023; Dale et al., 2022; Wechsler & do Nascimento, 2004).

The Wechsler Scales are further subdivided according to age group, comprising the Wechsler Intelligence Scale for Children (WISC) for the pediatric population and the Wechsler Adult Intelligence Scale (WAIS) for adults. Each edition of these instruments includes distinct subtests and tasks, along with specific standardizations that reflect linguistic, cultural, and national adaptations. Although strong or moderate correlations are generally observed among different versions of the scales, variability in performance outcomes has been documented in populations with autism spectrum disorder (ASD) or intellectual disability (ID). To date, there remains no definitive consensus regarding whether these instruments tend to underestimate, overestimate, or accurately capture the intellectual abilities of these groups (A. M. Nader et al., 2015; A.-M. Nader et al., 2016).

### 1.2. ASD and ID Cognitive Profile

A lack of consensus remains regarding the specific cognitive and diagnostic profiles derived from the Wechsler Scales (WS). Nonetheless, these instruments provide valuable information commonly associated with relevant clinical outcomes. Consequently, contemporary research employing WS aims to establish associations between their results and specific cognitive profiles (such as intellectual disability—ID), clinical impairments (such as deficits in adaptive behavior), and long-term functional outcomes (Sánchez-Izquierdo et al., 2023; Zemach et al., 2023). Within the spectrum of neurodevelopmental conditions, Full-Scale Intelligence Quotient (FSIQ) has been shown to correlate more strongly with reduced adaptive behavior and diminished performance in activities of daily living than in typically developing (TD) individuals (McQuaid et al., 2021). More specifically, individuals with autism spectrum disorder (ASD) who exhibit below-average IQ scores tend to demonstrate poorer adaptive behavior outcomes, as well as a marked discrepancy between intellectual performance and the ability to perform daily living activities (Tamm et al., 2022; Tassé & Kim, 2023).

From a complementary perspective, ID—characterized by reduced FSIQ and impaired adaptive functioning as defining diagnostic markers—consistently yields significantly lower scores across these metrics, along with notable deficits in executive functions (Erostarbe-Pérez et al., 2022; Spaniol & Danielsson, 2022). Furthermore, evidence indicates that individuals with ID exhibit considerable variability in FSIQ and index scores across subgroups, including those with mild or moderate ID. As previously mentioned, this disorder is notably heterogeneous, with studies often stratifying participants by FSIQ performance (Santegoeds et al., 2022).

Regarding the specific WS indices, ASD has been associated with greater impairments in Working Memory (WMI) and Processing Speed (PSI), typically resulting in approximately one standard deviation below the mean. Comparative studies between ASD and Attention-Deficit/Hyperactivity Disorder (ADHD) have demonstrated a more consistent intellectual profile in ASD, whereas ADHD does not exhibit a distinct or stable cognitive pattern (Kim & Song, 2020; Wilson, 2024). Additional research has identified subgroups within the ASD population that display comparatively higher Verbal Comprehension Index (VCI) scores and enhanced verbal intelligence (Calero et al., 2015; Chua & Xie, 2022; Litman et al., 2025).

On the other hand, individuals with ASD and intellectual impairment (ASD+ID) and those with ID appear to show a generalized pattern of deficits across cognitive indices, with no consensus regarding a comparative profile either among the indices themselves or between these groups (Mungkhetklang et al., 2016).

### 1.3. Meta-Analytic Framework for Cognitive Profiles in ASD, ID, and ASD+ID

Even with relevant data on both conditions, there is a lack of meta-analyses focused on the specific profile of non-syndromic intellectual disability (ID), particularly considering its Index variations rather than just Full-Scale IQ (FSIQ). Additionally, few studies attempt to compare autism spectrum disorder (ASD) and ID simultaneously, or to cross-analyze Wechsler Scale (WS) tests to identify this profile (Barnard, 2023; Dale et al., 2021).

Therefore, the present study proposes a meta-analysis that simultaneously examines ASD, ID, and ASD+ID across different versions of the main Wechsler scales (WAIS-III, WAIS-IV, WISC-IC, and WISC-V) to better understand the current literature on intelligence assessment in neurodevelopmental disorders. Specifically, this meta-analysis aims to:

1. Examine cognitive differences among individuals with ASD, ID, and ASD+ID, considering performance across indices and test types.
2. Compare the results obtained for these diagnoses against expected normative performance on the tests.
3. Compare the performance across different indices within each disorder.
4. Investigate moderators that may influence changes in cognitive profiles across distinct conditions.
5. Assess heterogeneity to better delineate the expressive variations found in ASD and ID.

## 2. Materials and Methods

### 2.1. Study Selection and Search Strategy

PsycNet, PubMed, Embase, and ScienceDirect databases were used for data collection. The strategy uses Boolean-based key terms, including: “ASD,” “IDD,” “ID,” “pervasive development,” “intellectual disability,” “asperger,” “autism spectrum disorder,” “WISC,” “WAIS,” “Wechsler Intellectual Profile,” adapting them to the specifications of each database. For a comprehensive overview of the search strategies and specific formulas, refer to the Supplementary Material, Figure S1. This research is registered with PROSPERO (2025 CRD420251038045) and used a 10-year time frame for the searches.

The “Patient Population, Interventions, Comparisons, and Outcomes” (PICO) model was selected to guide the selection and differentiation of data as follows (Eckstein et al., 2001):

- Participants (P): The study included individuals diagnosed with autism spectrum disorder (ASD) or Intellectual and Developmental Disabilities (IDD), including variations such as intellectual disability (ID) or Asperger’s Syndrome, as defined by the DSM-IV, DSM-V, or DSM-V-TR criteria. Articles involving ASD with FSIQ < 70 or those focusing on individuals with ASD and intellectual impairment were categorized as ASD+ID.
- Interventions (I): The focused intervention was the application of Wechsler’s scales, specifically WISC-IV, WISC-V, WAIS-III, or WAIS-IV. The studies were required to report FSIQ and cognitive index data explicitly.
- Comparison (C): Eligible studies included those that compared results using the instrument’s own normative data, a control group, or another mental health condition.
- Outcome (O): The primary outcomes were the cognitive indices derived from the tests. Inclusion in this phase required the presentation of all index scores from the respective test (FSIQ, VCI, PRI, WMI, and PSI, or FSIQ, VCI, FRI, VPI, and WMI in WISC-V).

Articles were excluded if they did not employ any of the selected tests, failed to report cognitive index data, or did not investigate ASD or ID. Additionally, only original empirical studies were accepted, and publications such as book chapters, conference presentations, brief reports, or review articles were not included. Studies for which full-text access or exact variable values were unavailable were also excluded from the analysis.

### 2.2. Data Extraction, Synthesis and Bias Assessment

The article screening and data extraction were conducted using Rayyan, a platform developed by the Qatar Computing Research Institute (QCRI) for systematic review management. Data collection was conducted on 23 May 2025, following a double-blind screening model involving five independent reviewers (GM, FN, MA, MO, and RS). The selection process was organized in two phases. In the first phase, MGA, MO, and RS independently screened one-third of the articles, focusing solely on the title, identification, and abstract to assess eligibility according to the PICO strategy. Meanwhile, GM reviewed all selected articles, serving as the double-blind reviewer responsible for cross-validation of initial decisions. In the second phase, full texts meeting the inclusion criteria were then reviewed in detail by GM, MA, MO, and RS according to predefined criteria. At each selection stage, at least two reviewers independently assessed the articles, and in cases of disagreement, FN provided additional analysis.

The following variables were extracted and organized in Microsoft Excel from the selected article: Author, Year, Country, Diagnosis, Intelligence Test Used, Number of Participants, Mean Age, Biological Sex (% Male), Diagnostic Criteria, Research Groups, Type of Study, and Index Scores (FSIQ, VCI, PRI, WMI, PSI, VPI, FRI). All included studies were then analyzed using the Strengthening the Reporting of Observational Studies in Epidemiology (STROBE) checklist, which comprised 22 items with multiple sub-items (Cuschieri, 2019). We considered studies to be at low risk of bias when at least 75% of the criteria are met. Results of the STROBE assessment are provided in Supplementary Material S1.

### 2.3. Data Synthesis

From each article, the following data were extracted: author, year, country, diagnosis, intelligence test used, number of participants, mean age, biological sex (% male), population diagnostic criteria, research groups, type of study, and cognitive indices (FSIQ, VCI, PRI, WMI, PSI, VPI, FRI). The primary variables included clinical diagnosis (ASD, ASD+ID, ID), assessment instrument (WISC-IV, WISC-V, WAIS-III, WAIS-IV), cognitive outcomes (FSIQ, VCI, PRI, WMI, PSI, VPI, FRI), and descriptive statistics (mean, standard deviation, and sample size).

### 2.4. Data Analysis

This meta-analysis was conducted using R (version 4.04) and the packages metafor, meta, tidyverse, openxlsx, and haven for data analysis and visualization. A continuous data model with random effects was applied to account for variations in test adaptations, populations, and standardizations. Effect sizes were converted into standardized mean differences (Hedges’ g), using a standardized control with a mean of 100 and a standard deviation of 15 to represent expected normative performance, as theorized by Cohen (1988). Effect magnitude was interpreted according to Cohen (1992), with g = 0.20 considered small, g = 0.50 medium, and g = 0.80 large.

Descriptive statistics (mean, standard deviation, and sample size) were collected and used to calculate standard error (SE = sd/√n) and variance (Var = SE^2^) for the sample groups in each study. Data with missing or zero variance were excluded. To assess heterogeneity, I^2^ statistical analyses were used and interpreted based on Cochrane guidelines: 0–40% may be unimportant, 30–60% may represent moderate heterogeneity, 50–90% substantial, and 75–100% considerable (J. Higgins et al., 2002; J. P. T. Higgins & Li, 2022). Heterogeneity was evaluated both for combined analyses across different tests, indices, and diagnoses, and more specifically, for diagnostic comparisons by test type and cognitive outcome (J. P. T. Higgins et al., 2020). Tau^2^ was calculated using the Restricted Maximum Likelihood (REML) method, with an unstructured covariance matrix and standard error adjustment via the Hartung-Knapp method (Röver et al., 2015). Finally, meta-regressions were conducted using categorical moderators (test type, diagnosis, and index by diagnosis), and subgroup analyses were performed by test type, diagnosis, and cognitive index. Finally, Sensitivity and Certainty analyses were performed and are available in Supplementary Material S4.

## 3. Results

**Figure 1:**
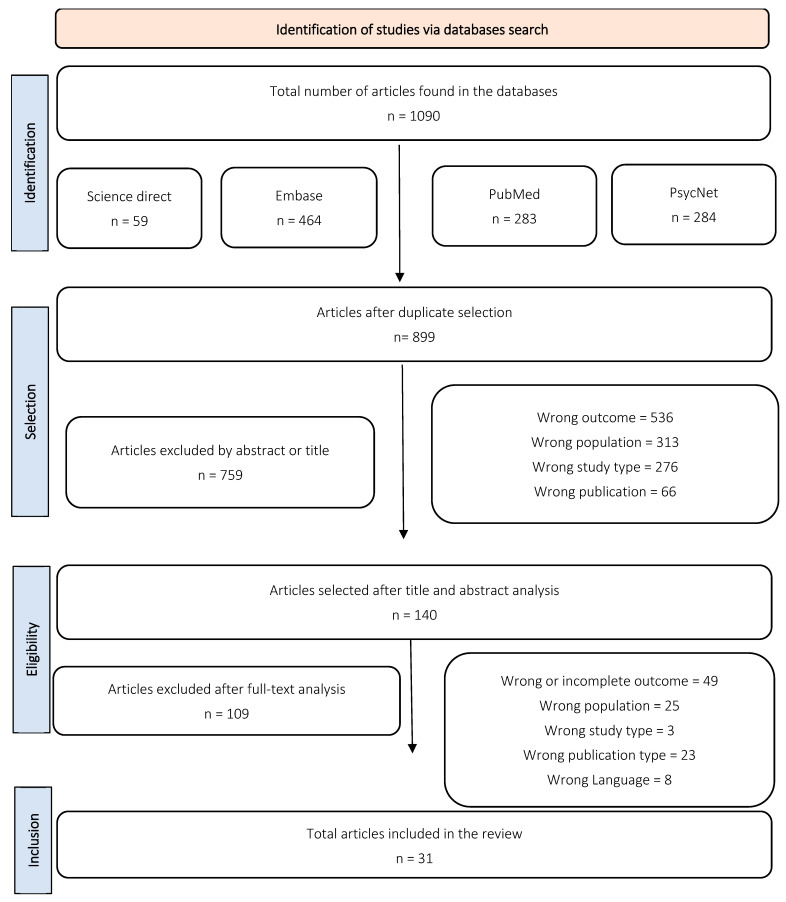
Selected articles’ flowchart detailing the screening process.

Initially, 1090 files were collected from the four databases (899 after duplicates were removed). Selection based on titles and abstracts yielded 140 articles; after full-text screening, 31 eligible studies were retained. Notably, several tests included more than one distinct sample group (e.g., cases stratified by FSIQ ranges or other distinguishing characteristics). Figure 1 presents the PRISMA flowchart detailing the screening process.

### 3.1. Population Characteristics

Table 1 presents the sociodemographic data for the selected articles, including country, diagnosis, intelligence test used, number of participants, average age, gender distribution, research group, study type, and exclusion criteria.

Most of the study samples consisted predominantly of males: 16 studies with ≥80% male participants and 15 with 50–80%. The cognitive assessments performed were the WISC-IV (*n* = 24), followed by the WAIS-IV (*n* = 7), WAIS-III (*n* = 4), and, lastly, the WISC-V (*n* = 3). Remarkably, a significantly higher number of research samples included ASD (k = 28) than ID (k = 08) or ASD+ID (k = 3); the repercussions of this will be addressed in the Section 4.

Most studies originated from Europe (Italy, France, Spain, the UK, Sweden, and Germany), North America (the USA and Canada), and Asia (Japan, South Korea, Hong Kong, and China), with additional research conducted in Brazil, Israel, and Iran. Most research followed observational or cross-sectional designs and commonly applied test-based inclusion criteria. Among these, the autismautism Diagnostic Observation Schedule (ADOS) was most frequently used for autism spectrum disorder (ASD) diagnosis, followed by the autismautism Diagnostic Interview-Revised (ADI-R) and Social Responsiveness Scale (SRS); for intellectual disability (ID), the Wechsler scales were employed, using FSIQ < 70 as the threshold.

For database construction, studies involving High-Functioning autismautism spectrum disorder (HFASD), Asperger’s Syndrome (AS), and ASD were grouped under the ASD category, considering that it includes autism spectrum conditions without intellectual impairment. Conversely, studies on ID and intellectual disabilityintellectual development disorder (IDD) were categorized together, excluding those focused predominantly or specifically on syndromic samples.

### 3.2. Descriptive Data

Table 2 presents the basic descriptive results of the research, including the mean (with its standard deviation), the number of sample groups (k), the standardized mean difference, and the number of participants per category.

The results in Table 2 revealed distinct cognitive profiles between ASD and ID individuals. In the ASD group (aggregated sample: 553–2024 participants), the mean FSIQ was 95.00 (SD = 9.94; k = 28). Specific indices showed varied patterns: PRI had the highest mean (100.2; SD = 9.94), followed by VCI (98.66; SD = 12.27). In contrast, PSI (88.14; SD = 8.57) and WMI (92.29; SD = 8.76) yielded the lowest scores. Additionally, VPI (101.4; SD = 4.02) and FRI (100.7; SD = 4.29) were close to the normative value of 100, although based on fewer studies (k = 3).

For the ID group (*n* = 335; k = 7), the results were lower than those for ASD, consistent with the diagnostic classification. The FSIQ averaged 55.35 (SD = 1.93), which is the lowest score. WMI (59.81; SD = 3.39) and VCI (61.18; SD = 0.64) showed the lowest performance, while PRI (66.98; SD = 5.33) and PSI (64.71; SD = 1.85) were comparatively higher.

In the ASD+ID subgroup (*n* = 102; k = 3), FSIQ (53.35; SD = 1.93) and WMI (59.81; SD = 3.39) were below 60. VCI (61.18; SD = 0.64), PSI (64.71; SD = 1.85), and PRI (66.98; SD = 5.33) exceeded 60 but remained within the intellectual impairment range (<70).

### 3.3. Comparisons Between Tests and Diagnosis

**Figure 2:**
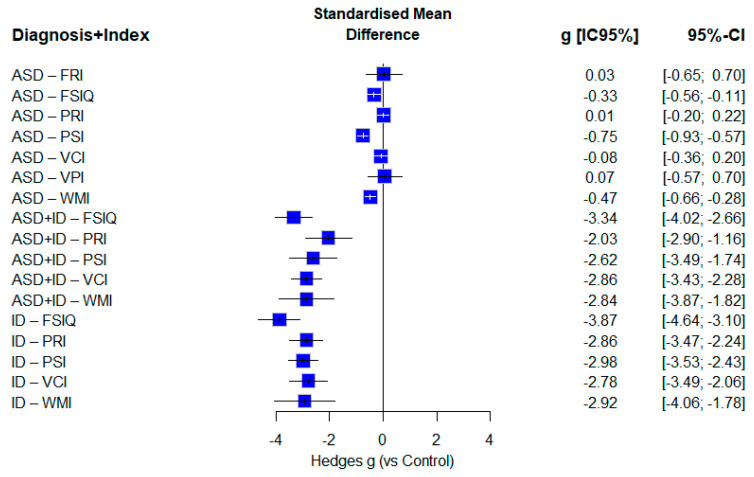
Forest plot indicating standardized mean differences (g) associated with the combination of diagnostic and index subgroups. Blue cubes with black or white confidence intervals represent all effects. The overall effect size was not included as this analysis combined subgroups. The 95% confidence interval and mean difference are shown on the right side of the plot.

The standardized mean difference test, Hedge’s g, was performed, considering a standardized control sample with a mean of 100 and a standard deviation of 15 for the test, as theorized by Cohen (1988). Figure 2 evaluated performance based on diagnosis and cognitive index using the standardized mean deviation strategy (Hedges’ g), accounting for random effects and providing 95% CIs.

In the ASD group, effect sizes were predominantly small to moderate in magnitude. The largest was observed in PSI-ASD (g = −0.75), classified as large, followed by a moderate effect in WMI-ASD (g = −0.48). Other indices showed small or negligible effects: FSIQ-ASD (g = −0.34), VCI-ASD (g = −0.07), FRI-ASD (g = 0.03), PRI-ASD (g = 0.01), and VPI-ASD (g = 0.07).

In contrast, all ID and ASD+ID values had large and negative effects. The smallest reduction was in PRI-ASD+ID (g = −2.03), while the most pronounced effects were in FSIQ-ASD+ID (g = −3.34) and FSIQ-ID (g = −3.87), indicating the most significant divergence from the control groups. ID indices were consistently high: PRI-ID (g = −2.86), PSI-ID (g = −2.98), VCI-ID (g = −2.78), and WMI-ID (g = −2.92), all within overlapping confidence intervals, reinforcing a homogeneous deficit profile.

**Figure 3:**
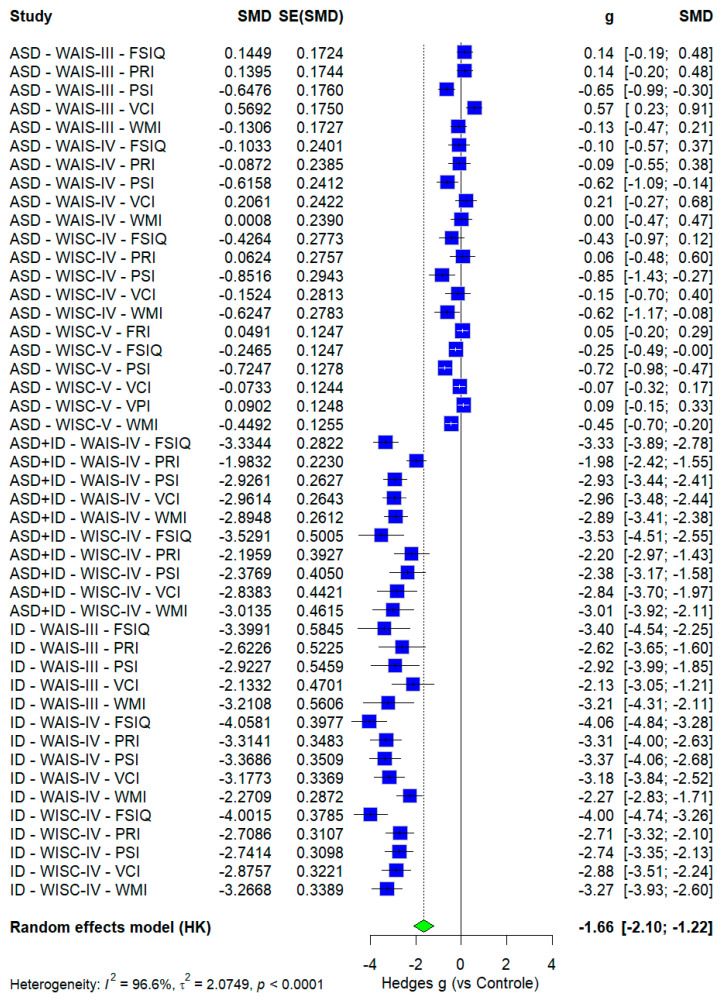
Forest plot of standardized means (g) using three simultaneous groupings: test type, cognitive index, and diagnosis. Blue cubes with black or white confidence intervals represent all effects, *the green diamond and associated dashed line indicate the pooled effect for outcomes, test, and diagnostic, based on a random-effects model*. The 95% confidence interval and mean difference are shown on the right side of the plot.

The ASD+ID group mirrored the ID profile but showed wider confidence intervals. The ascending order of effects was: PRI (g = −2.03), PSI (g = −2.62), VCI (g = −2.86), and WMI (g = −2.84). Additionally, standardized mean differences (Hedges’ g) were calculated across subgroups by test type, cognitive index, and diagnosis (Figure 3).

Stratified results by test type revealed small to moderate effects in the ASD group. In the WAIS-III, effects were predominantly small—FSIQ (g = 0.14), PRI (g = 0.14), WMI (g = −0.13), VCI (g = 0.57)—with moderate reduction in PSI (g = −0.62). In the WAIS-IV, effects ranged from small, PRI (g = −0.09), VCI (g = −0.15), WMI (g = 0.00), to moderate in PSI (g = −0.62). The WISC-IV showed a similar pattern, with small effects in FRI (g = 0.05), VCI (g = 0.07), VPI (g = 0.09), and PRI (g = 0.06), but more pronounced reductions in PSI (g = −0.72) and WMI (g = −0.45).

In the ASD+ID group, all effects were large and negative. WAIS-IV scores showed negative substantial magnitudes: FSIQ (g = −3.34), PRI (g = −1.93), PSI (g = −2.93), VCI (g = −2.96), and WMI (g = −2.89). WISC-IV results followed a similar profile, with even stronger effects: FSIQ (g = −3.53), VCI (g = −2.88), WMI (g = −2.38), PRI (g = −2.38), and PSI (g = −2.84).

For the ID group, consistently large effects were observed across all test versions. In the WAIS-III, PSI (g = −2.13) and WMI (g = −2.12) stood out. WAIS-IV showed even greater deficits: FSIQ (g = −4.06), PRI (g = −3.43), PSI (g = −3.37), VCI (g = −3.17), and WMI (g = −2.27). WISC-IV results remained elevated: FSIQ (g = −4.00), PRI (g = −2.71), VCI (g = −2.88), and WMI (g = −3.27).

### 3.4. Meta-Regressions

Two meta-regressions were performed using a categorical model to examine the effects of diagnosis or test type on cognitive outcomes. Table 3 presents the meta-regression results, which used diagnosis type as a moderator.

ID (β = 67.50; SE = 2.29; Z = 29.40; *p* < 0.001; 95% CI = 63.00–72.00) was selected as the reference intercept, as intellectual impairment is a diagnostic criterion in multiple studies. Compared to ID, the ASD group showed a significant positive difference in the coefficient (+26.01; SE = 2.55; Z = 10.20; *p* < 0.001). In contrast, the ASD+ID group did not differ significantly from ID (β = −4.67; SE = 2.66; Z = −1.75; *p* = 0.080; 95% CI = −9.89–0.55).

Additionally, a meta-regression model was applied using test type as a moderating variable (Table 4).

The WAIS-III was used as the moderator in the meta-regression model, yielding a coefficient of β = 82.88 (SE = 4.33; Z = −0.27; *p* = 0.70; 95% CI = 76.28–89.47). No other test showed a statistically significant difference when compared to WAIS, regardless of version or age range. WAIS-III was selected as the intercept because it demonstrated the highest mean performance among all tests. Finally, a regression analysis was conducted that combined diagnosis and cognitive index simultaneously (as shown in Table 5).

In this model, the ASD group showed a significant difference only in FSIQ, PSI, and WMI compared to FRI (moderator and index values close to the norm). However, analyzing the ASD+ID group, only PRI showed a significant difference with FSIQ (moderator and with the lowest result). Finally, in the ID group, all indexes (PSI, PRI, VCI, and WMI) showed a statistically significant difference from FSIQ (the lowest result).

### 3.5. Heterogeneity

**Figure 4:**
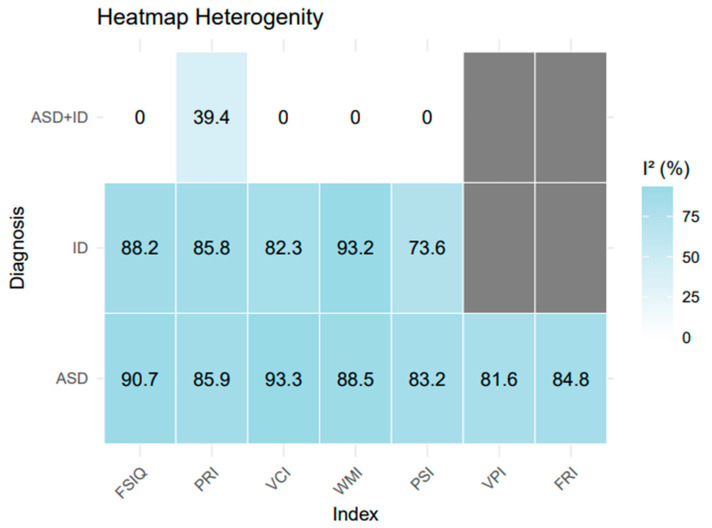
Heterogeneity heatmap. Note: Gray blocks indicate missing data for VPI and FRI in the ASD+ID and ID groups.

An I^2^ heterogeneity test (Huggies and Thompson) was also performed to assess the degree of heterogeneity in the study sample. Figure 4 displays the I^2^ values in a heatmap from the iteration covering diagnosis and outcomes.

In the ASD group, all heterogeneity values were classified as substantial (I^2^ > 75%). In ascending order, the heterogeneity rates were: PSI (83.2%), VPI (81.6%), FRI (84.8%), PRI (85.9%), WMI (88.5%), FSIQ (90.7%), and VCI (93.3%). In the ID group, PSI (73.6%) was classified as moderate (<75%), while FSIQ (88.2%), PRI (85.8%), VCI (82.3%), and WMI (93.2%) presented high to very high values. In ascending order: PSI (73.6%), VCI (82.3%), PRI (85.8%), FSIQ (88.2%), and WMI (93.2%).

In the ASD+ID group, heterogeneity was null (I^2^ = 0) for nearly all outcomes, except for PRI (39.4%), which was classified as low or moderate depending on the analysis. This lack of heterogeneity is attributed to the limited number of available studies (reduced k). Finally, there were no results for IPV or FRI in the ASD+ID and ID groups, which were treated as non-existent data and represented in gray in the graph.

## 4. Discussion

The study identified distinct profiles for autism spectrum disorder (ASD) and intellectual disability (ID), with exploratory data for ASD+ID. While ASD and ID profiles showed significant differences, no differences were found between ID and ASD+ID. No significant variation emerged across versions of the Wechsler scales, and heterogeneity levels were moderate to high for ASD and ID.

### 4.1. Categorization and Description

A key observation was the predominance of data collection in countries with higher Human Development Index (HDI) or socioeconomic status, with limited research from Latin America, Africa, the Middle East, and Oceania. Other data also indicate that countries in the Global North have better access to evidence-based treatments and cognitive testing for neurodevelopmental conditions. Furthermore, there are signs of underrepresentation of ethnically and politically marginalized groups, underscoring a gap in the current literature. Notably, some studies failed to report racial demographics of their samples (Huang et al., 2020; Rosen et al., 2021).

Geographically, most studies were conducted in university hospitals, specialized centers, or mental health clinics. In contrast, research from lower-income countries was less frequent, possibly reflecting limited resources and reduced availability of specialized services. This discrepancy highlights a gap in the literature regarding ASD and ID data from less developed regions (Lakens, 2022). This lack of knowledge from countries in LAMIC may limit analysis due to genotypic differences and regional peculiarities such as reduced access to inclusive educational and health practices (especially in poorer regions), along with cultural and regional differences (e.g., varying levels of linguistic complexity and approaches in social comunication), or even divergent public health policy strategies (Girianelli et al., 2023; Sukiennik et al., 2022).

Another challenge is the difficulty in isolating ASD from ID due to frequent neurodevelopmental comorbidities. Few studies excluded other conditions beyond the target diagnosis, hindering the development of more accurate profiles, as many studies repeatedly analyzed mixed populations of Attention Deficit Hyperactivity Disorder (ADHD), ASD, and/or ID (Bougeard et al., 2021; Wilson, 2024).

Finally, regarding sample characterization, although a male predominance in ASD and ID groups was expected, some studies reported samples composed almost entirely of males (90–100%). This skewed gender discrepancy raises concerns about the underrepresentation and potential invisibility of pervasive disorders in females, who—despite genotypic and statistical differences—can still present the condition with distinct symptomatic and cognitive profiles, including on Wechsler scales (Calderoni, 2023; McQuaid et al., 2021). Previous research has demonstrated significant sex-based differences in intellectual functioning, with women often achieving higher scores on the VCI and PSI but lower scores on the PRI compared to men (Giofrè et al., 2024; McQuaid et al., 2021). The predominance of male samples in the present review—drawn mainly from Europe, Asia, and North America, and with a greater proportion of children—further limits the generalizability of findings (Di Salvo, 2024).

### 4.2. Analysis Between Diagnoses and Tests

#### 4.2.1. Analysis Between Diagnoses

Clear cognitive profiles were identified relative to the normative sample, across all diagnoses and within each cognitive index. ASD consistently demonstrated the lowest performance in the Processing Speed Index (PSI; g = −0.76; 95% CI: −0.92 to −0.60), followed by the Working Memory Index (WMI; g = −0.48; 95% CI: −0.67 to −0.29). These findings align with prior autism literature, which frequently reports deficits in processing speed and working memory. Notably, this pattern emerged across all versions of the Wechsler scales, particularly for PSI, consistent with other studies of ASD profiles (Kim & Song, 2020; Wilson, 2024).

In this study, individuals with ASD consistently performed worse in processing speed compared to typically developing (TD) individuals. This slower processing speed appears to be a stable impairment pattern across different cognitive tasks in ASD (Möde et al., 2023; Wu et al., 2018), indicating difficulties in perceiving, processing, and responding to stimuli. However, PSI tasks also involve other cognitive processes such as attention, inhibitory control, and motor coordination (Zapparrata et al., 2023), which may contribute to the observed impairments. Several studies have found longer reaction and performance times in distinct cognitive tasks, even in contexts where the success rate and quality of the task are not impaired, which corroborates the hypothesis of stability of WMI and PSI impairments in ASD across different assessments (Rabiee et al., 2019; Wu et al., 2018; Zapata-Fonseca et al., 2018; Zapparrata et al., 2023). Some authors hypothesize that this difficulty may be associated with the weak central coherence hypothesis in ASD, leading to slower responses due to difficulties integrating information into a coherent Gestalt (Bojda et al., 2021).

From a neurobiological perspective, consistent findings of reduced corpus callosum (CC) volume and altered structural connectivity, together with increased overall brain volume, have been strongly linked to deficits in attention, executive functions, and working memory (WMI) (Bojda et al., 2021; Sacco et al., 2015; Valenti et al., 2020). These alterations may partly explain the cognitive profiles observed in individuals with ASD. Additional studies have reported differences in connectivity in regions such as the lateral occipital cortex (LOTC), the fusiform gyrus (FG), the temporal lobe, and the insula, which are associated with ASD-related symptoms and cognitive impairments (Eyler, 2018; Delbruck et al., 2019). Taken together, these findings reinforce the neurobiological basis of ASD while underscoring the heterogeneity of structural and functional brain differences that may contribute to variability in cognitive outcomes.

ID data revealed a significantly more pronounced and consistent reduction in impairment across the Wechsler scales (Figure 2). These generalized deficits are historically associated with ID, though performance differences across indices were relatively narrow—except for FSIQ, which showed significantly lower scores. Meta-regression and plot-intercept analyses confirmed this discrepancy between FSIQ and other indices. It is hypothesized that intelligence distribution tends to be more variable at the extremes compared to individual cognitive indices, as supported by prior studies (Toffalini et al., 2019; Tuon et al., 2023; Wechsler & do Nascimento, 2004). From a neurophysiological perspective, intellectual impairment is associated with disproportionate alterations in Gray Matter Volume (GMV) and White Matter Volume (WMV), thereby impacting functional connectivity across various brain regions. Specifically, the dorsal medial prefrontal cortex (dmPFC), bilateral orbital part of the inferior frontal gyrus (orb_IFG.L, orb_IFG.R), right cuneus (cuneus.R), and bilateral middle frontal gyrus (MFG.L, MFG.R) show significant effects. Negative correlations between GMV malformation and FSIQ further reinforce the structural basis of intellectual deficits in ID (Ma et al., 2021)

Regarding the co-occurrence of ASD and ID, results revealed a distinct impairment profile on Wechsler scales, more closely resembling ID than ASD. Research addressing all WS indices in ASD+ID is scarce; however, the available data indicate significant intellectual deficits in this population (Santambrogio et al., 2023). Figure 2 reinforces this pattern, with most ID indices falling within the range of their ASD+ID counterparts. Meta-regression also failed to differentiate between ASD+ID and ID profiles, strongly reinforcing their similarity. This similarity suggests that ID presence in comorbid cases exerts a more substantial influence on intelligence profile (cognitive performance), further supporting models that include ID and ASD+ID within the same response pattern on Wechsler scales. Two hypotheses emerge: first, ID to mask the cognitive presence of ASD, and, second, the ASD+ID profile is effectively similar to ID, reinforcing the theory of genotypic-phenotypic differences between ASD+ID and ASD (Genovese & Butler, 2023; Litman et al., 2025). Complementary eye-tracking data have identified distinct response profiles in ID and ASD+ID, reinforcing the thesis that these are separate profiles but with similar intellectual characteristics in standardized testing (Lian et al., 2023). These findings highlight the need for comprehensive assessments—including multiple tests and analytic strategies—to differentiate ASD, ID, and ASD+ID outcomes. Notably, when the diagnosis type was moderated (with ID as the intercept), no significant differences were found between ID and ASD+ID. This finding represents a novel contribution of the present review, as no prior studies have conducted this type of analysis using Wechsler scales.

#### 4.2.2. Analysis Between Tests

Figure 3 presents the profile based on the subgroup combination of outcome, diagnosis, and test type. No significant differences were found between test, a finding corroborated by the forest plot, which showed that the outcome+diagnosis+test combinations fell within the confidence intervals of their counterparts across different test categories (e.g., ASD–WAIS-III–FSIQ: g = 0.14; −0.19 to 0.48; ASD–WISC-IV–FSIQ: g = −0.43; −0.97 to 0.12). This consistency suggests stability and reliability across current versions of the Wechsler scales when dealing with identical constructs, likely due to the maintenance of core subtests such as matrix reasoning, cubes, and vocabulary during updates (Wilson, 2024).

Table 3 confirmed this lack of differentiation through meta-regression using WAIS-III as the moderator. However, these results contrast with prior literature reporting differences between WISC-IV and WISC-V, and even between WAIS-IV and WISC-V—particularly in indices such as VCI (Kuehnel et al., 2019; Stephenson et al., 2021; Wilson, 2024). For example, Kuehnel et al. (2019) observed differences in VCI between WISC-IV and WISC-V, with a slight difference in the Composite score (5.08) in a longitudinal study. In contrast, A. M. Nader et al. (2015) found more pronounced differences between WISC-III and WISC-IV, particularly in the fourth version’s PRI and VCI. Nonetheless, findings remain inconsistent, with no consensus across studies (Stephenson et al., 2021; Wilson, 2024).

Some possibilities may explain the profile measured in this review. The meta-regression method may have limited sensitivity in groups with high heterogeneity, significant standard errors, or deviations, making it difficult to detect subtle differences. Moreover, comparing Figure 3 with Supplementary Figure S9, aggregated mean values indicated more pronounced differences, whereas standardized mean differences (g) showed no significant effect. These contrasting results indicate that, even when raw scores vary, the effect sizes of ASD, ID, or ASD+ID across test versions do not differ sufficiently from TD performance to overcome sample heterogeneity (Cohen, 1992, 1988).

Finally, unlike PSI, WMI scores were closer to the normative range, with WAIS-IV and WAIS-III showing no significant deviations from the mean. This disparity raises two hypotheses: first, that WAIS tests may have lower sensitivity for detecting working memory deficits; second, that such deficits in ASD are more detectable in childhood and diminish in adulthood. Supporting this, Demetriou et al. (2018) found, in their meta-analysis, developmental differences in working memory among individuals with ASD, with more potent effects in children under 12, reduced effects in adults, and no significant differences in adolescents (Ceruti et al., 2024).

### 4.3. Heterogeneity and Bias

Regarding heterogeneity (Figure 4), ASD presented considerable variability across all outcomes. While this heterogeneity may influence the results, it was anticipated—especially in comparison to other meta-analyses focused on cognitive processes in autism, where IQ differences between samples are not controlled (as in the present analysis, which explicitly analyzes these differences) (Ceruti et al., 2024; Demetriou et al., 2018). For ID, heterogeneity was also substantial, reflecting the broad variability in cognitive and symptomatic profiles within the intellectual disability spectrum, which includes subcategories stratified by FSIQ levels (e.g., moderate vs. mild intellectual disability). Elevated I^2^ values confirmed this heterogeneity for both ASD and ID.

Even when test type was included as a moderator, ASD and ID continued to show high heterogeneity (as detailed in the Supplementary Material). This result suggests that test type variation alone cannot fully account for the observed heterogeneity (see Supplementary Table S1). Contributing factors likely include the diversity of subtypes, variations in these neurodevelopmental conditions, regional differences in test administration (e.g., language differences and statistical differences in validation), and difficulties controlling for comorbidities in the included studies. Similar findings have been reported in previous reviews (J. P. T. Higgins & Li, 2022; Huang et al., 2020; Wilson, 2024).

In contrast, ASD+ID results indicated low or no heterogeneity, consistent with the minimal variation in mean differences and confidence intervals across the studies (Supplementary Figures S1–S5). However, these results are strongly influenced by the limited number of studies (k = 3), reducing the reliability of generalizations. Thus, ASD+ID data should be interpreted as exploratory, while ASD and ID findings carry greater relative weight (J. P. T. Higgins & Li, 2022*)*.

Final point: due to the limited sample size, only ASD (excluding VPI and FRI) was included in funnel plot analysis, with results detailed in the Supplementary Material S3. Overall, these findings highlight the need for caution when generalizing across disorders, given their high variability and individual differences, and emphasize that diagnosis and intervention require individualized, context-sensitive clinical analyses.

### 4.4. Clinical Relevance

Impairments in Working Memory Index (WMI) and Processing Speed Index (PSI) in individuals with autism have significant implications for outcomes such as academic achievement, workplace functioning, daily living activities, and adaptive behavior. These deficits are consistently associated with challenges in ASD, even when overall IQ does not differ from that of typically developing (TD) individuals (McQuaid et al., 2021; Rommelse et al., 2020).

In intellectual disability (ID), deficits in Full Scale IQ (FSIQ) and other cognitive indices predict moderate to poor outcomes in education, adaptive behavior, and quality of life—even within ID severity gradations, where the degree of impairment varies according to FSIQ levels. While increases in IQ are associated with improvements in adaptive behavior, the reverse is not necessarily true: gains in adaptive functioning do not correspond to increases in FSIQ in most cases (Dakopolos et al., 2024; Tassé & Kim, 2023).

This study confirmed the hypothesized cognitive profiles for ASD and ID, demonstrating their consistency across diverse samples and test versions. These findings support the use of the Wechsler scales for identifying such deficits regardless of the applied version. However, the data also revealed that the Wechsler scales have limited sensitivity in distinguishing autism with intellectual deficit within ID samples. Therefore, while valuable for assessing both conditions, the Wechsler scales should be complemented by instruments that more precisely capture the autistic profile within ID, such as caregiver-report measures and ASD symptom-based assessments (e.g., SRS-2, VABS-III, ADOS) (Yu et al., 2024).

It is important to emphasize that both ASD and ID remain clinical diagnoses. ASD benefits more from index-based assessment given its specific functional profile, whereas ID groups often show similar scores across indices, except for FSIQ. Consequently, neither intelligence scores nor behavioral rating scales alone are sufficient for comprehensive diagnostic conclusions. In addition to symptom scales and cognitive tests, clinical interviews with caregivers, family members, or close associates, as well as direct clinical observation, are essential. The main differentiating factor for ID with or without ASD comorbidities is the presence of social symptoms and behavioral restrictions (e.g., echolalia, stereotypies). For ID, assessment of comprehension—particularly in pragmatic and conceptual domains—is critical, given the shared social impairments across both conditions (Braconnier & Siper, 2021; Carreiro et al., 2017; Yu et al., 2024).

### 4.5. Limitations

Despite consistent findings, several limitations of this review warrant clarification. First, notable sampling biases were identified in the selected studies. There was a predominance of male participants and a concentration of research conducted in North America, Western Europe, and Asia, with limited representation from Latin America, Africa, and the Middle East—highlighting a gap in the current state of the literature.

Another relevant point is that many studies were conducted in specialized clinics, often relying on convenience sampling without formal sample size calculations. This sampling method raises concerns about the representativeness of the samples relative to the general population, which should be considered when interpreting the results. Moreover, many studies did not control for comorbidities, including various neurodevelopmental disorders within ASD or ID participant groups.

Few studies included ASD+ID samples, and it was not possible to meta-analyze subtests due to a methodological preference for studies reporting all index scores, rather than all the subtests that comprise them (Lakens, 2022; Noordzij et al., 2011). These characteristics of literature, along with the present meta-analysis, should be carefully considered when generalizing the findings.

## 5. Conclusions

This review identified consistent patterns of cognitive performance, with relative weaknesses in Working Memory Index (WMI) and Processing Speed Index (PSI) among individuals with ASD, and more pronounced impairments—particularly in Full Scale IQ (FSIQ)—in both ID and ASD+ID groups. These patterns persisted regardless of the Wechsler scale version used, with no statistically significant differences in performance across test editions. Notably, the ASD+ID group did not differ significantly from the ID group but showed marked differences compared to the ASD group. However, few studies specifically addressed the ASD+ID comorbidity, so further studies are necessary to confirm this finding.

Future research should expand the investigation of ASD+ID and ID using the Wechsler scales to enhance understanding of their cognitive profiles. Greater precision in sample size estimation is also strongly recommended, along with increased research efforts in underrepresented regions of the Global South—particularly Latin America, Africa, and the Middle East. Additionally, whenever possible, improved control of comorbidities within samples is encouraged to strengthen variable control and the interpretability of findings. Finally, research analyzing other key variables in understanding ASD and ID, such as adaptive behavior, social cognition, and Executive Functions, is also strongly recommended.

## Figures and Tables

**Table 1:** Study samples’ sociodemographic categorization.

ID	Author (Year)	Country	Diagnosis	Test	N	Mean Age (SD)	Sex (%M)	Diagnostic Criteria	Research Groups	Research Type	Exclusions/Additional Criteria
1	Baum et al. (2015)	USA	ASD	WISC-IV	40	12.91 (1.91)	87.50%	ADOS, multidisciplinary clinical assessment	ASD (WISC-IV or SB-5)	Observational	Unable to meet test, genetic or metabolic disorder, substance use
2	Calero et al. (2015)	Spain	AS	WISC-IV	45	9.61 (1.45)	89%	Previous diagnosis	AS (High Scores, Non Leaners, Leaners)	Observational	Other disorders; unconfirmed diagnosis
3	Jin et al. (2023)	China	HFASD	WISC-IV	128	6.8	88.30%	DSM-5, ABC, medical evaluation	HFASD, DSLDs, TD	Transversal	FSIQ > 70, other neurological disease
4	Kim and Song (2020)	South Korea	ASD	K-WISC-IV	49	8.47 (2.95)	100%	K-WISC-IV, interviews, scales (SRS, CARS)	ASD, ADHD	Retrospective	FSIQ < 70; neurological comorbidities
5	Levine et al. (2024)	USA	ASD	WISC-IV/V	214	9.8 (1.55)	82.44%	ADOS-2, ADOS-G, K-SADS	ASD (WISC IV/V), TD	Transversal	FSIQ ≥ 80; no genetic comorbidities
6	G. Li et al. (2017)	China	HFASD	WISC-IV	32	10.31 (3.34)	100%	DSM-5 (supervised MD/PhD)	HFASD, ADHD, TD	Transversal	FSIQ > 7 0
7	W. Li et al. (2024)	China	ASD	WISC-IV	257	7.55 (1.59)	85.21%	DSM-5 (medical team)	ASD (school/non-school)	Transversal	Native Mandarin speakers
8	Linnenbank et al. (2022)	Germany	ASD	WISC-IV	101	10.63 (2.72)	90.10%	DSM-5 (specialized team)	ASD FSIQ ≥ 100, FSIQ ≤99	Transversal	FSIQ > 70; fluent language, achieve ADOS and ADI-R
9	A. M. Nader et al. (2015)	Canada	ASD/AS	WISC-IV	66	10.6 (2.7) and 11.5 (3.2)	96% and 80 (ASD/AS)	ADI-R/ADOS-G, DSM-IV	ASD, AS, TD	Transversal	FSIQ < 70; genetic syndromes
10	A.-M. Nader et al. (2016)	Canada	ASD	WISC-IV	25	11.0 (2.8)	96%	DSM-IV-TR, ADI-R/ADOS-G	ASD vs. TD	Comparative	No genetic/neurological comorbidities
11	Operto et al. (2021)	Italy	HF-ASD	WISC-IV	19	8.84 (2.36)	68%	ADOS-2, ADI-R	HF-ASD, ADHD, SLD, TD	Transversal	FSIQ < 70; neurological comorbidities
12	Peñuelas-Calvo et al. (2021)	Spain	AS	WISC-IV	84	11.64	92.90%	DSM-IV + ADI-R/ADOS-G	AS	Transversal	FSIQ ≥ 70
13	Rabiee et al. (2019)	Iran	ASD	WISC-IV	43	11	90%	DSM-5 + ADI-R/ADOS-G + GARS-2	ASD (IQ matched/non-matched), TD	Transversal	No neurological/sensory comorbidities
14	Toffalini et al. (2019)	Italy	ID	WISC-IV	198	12.47 (2.56)	68%	DSM-5 + Vineland-II/ABAS-II	ID Mild (FSIQ < 70), Moderate (FSIQ < 55)	Transversal	FSIQ < 70
15	Tuon et al. (2023)	Brazil	ID	WISC-IV	16	10.76	55.90%	FSIQ < 75 + clinical evaluation	ID vs. TD	Case–control	6–16 years; no neurological diagnoses
16	Mungkhetklang et al. (2016)	Australia	ID	WISC-IV	8	14	65.20%	DSM-IV	ID without ASD (*n* = 15), ID+ASD (*n* = 8)	Transversal	FSIQ ≤ 70
17	Erostarbe-Pérez et al. (2022)	Spain	IDD	WISC-IV	83	15	55.40%	WISC-IV (TIQ 45–84)	DI, FIL	Transversal	FSIQ 45–84; 11–18 years
18	Audras-Torrent et al. (2021)	France	ASD	WISC-V	121	10.7	84.30%	ADOS-2, ADI-R, WISC-V, VABS-II	Subgroups by cognitive profile	Transversal	FSIQ > 70; no sensory impairments
19	Stephenson et al. (2021)	USA	ASD	WISC-V	349	10.5	82.80%	Clinical evaluation	ASD	Factor validation	No additional criteria
20	Kanai et al. (2017)	Japan	ASD	WAIS-III	120	29.6 (8.3)	78.30%	DSM-IV-TR, ADOS-2, PARS, DISCO	ASD, ADHD	Transversal	IQ ≥ 70; no psychiatric comorbidities
21	Marinopoulou et al. (2016)	Sweden	ASD	WAIS-III	50	27.7 (3.9)	50%	DSM-IV (psychiatrists/psychologists)	Asperger’s, Schizophrenia	Transversal	No explicit criteria
22	Zemach et al. (2023)	Israel	IDD	WAIS-III	100	34.53–63.47 *	Variable	Previous diagnosis	IDD (by age group: 30–69 years)	Transversal	No additional criteria
23	Lifshitz et al. (2018)	USA	IDD	WAIS-III	31	31.14 (5.84)	N/A	DSM-5, WAIS-III, verbal tests	ID (full inclusion/adapted course)	Transversal	No severe maladaptive behaviors
24	Cicinelli et al. (2022)	Italy	ASD	WAIS-IV	229	26.3 (9.35)	75%	DSM-5, ADI-R, ADOS-4, RAADS	ASD Level 1 and 2	Transversal	No additional criteria
25	Eyler (2018)	France	ASD	WAIS-IV	27	28 (9.4)	100%	DSM-5, ADI-R, ADOS	ASD and TD	Transversal	FSIQ > 70
26	Erickson et al. (2020)	USA	ID	WAIS-IV	62	35.27 (12.22)	54.80%	DSM-5	ID	Observational	Previous diagnosis
27	Leung et al. (2019)	Hong Kong	ASD	WAIS-IV	23	20.09 (3.32)	87%	DSM-IV-TR/5 (psychiatrists/psychologists)	ASD	Transversal	FSIQ > 70; native speakers
28	Tse et al. (2019)	United Kingdom	ASD	WAIS-IV	28	61	78.60%	Self-report + clinical confirmation	ASD	Transversal	Age ≥ 50 years; IQ ≥ 70
29	Bucaille et al. (2016)	France	ASD	WAIS-IV	16	26.6	68.75%	ICD-10, ADI-R	AS and TD	Comparative	FSIQ > 70; diagnosis in adulthood
30	Santambrogio et al. (2023)	Italy	IDD	WAIS-IV	120	57	75%	SPAIDD-G, STA-DI, clinical records	IDD	Transversal	DSM-5-TR
31	Giofrè et al. (2019)	Italy	ASD	WISC-IV	50	12.9	82%	Clinical information, Qi test	ASD and ASD+ID	Transversal	Diagnóstico prévio

ACRONYMS: ASD = autism spectrum disorder; ID = Intellectual disability; IDD = intellectual disabilityintellectual development disorder; TD = Typically Development; ADOS = autismautism Diagnostic Observation Schedule; ADI-R = autismautism Diagnostic Interview-Revised; HFASD = High-Functioning autismautism spectrum disorder; AS = Asperger’s Syndrome; ADHD = Attention Deficit Hyperactivity Disorder; SLD = Specific Learning Disorder; VABS-II = Vineland Adaptive Behavior Scales; DSM = Diagnostic and Statistical Manual of Mental Disorders. Note: * Zemach et al. (2023) divided the groups into 10-year intervals (30–39, 40–49, etc.).

**Table 2:** Sample descriptive data.

ASD	*n*	k	M (SD)	SMD
FRI	553	3	100.7 (4.29)	0.03
FSIQ	2024	28	95.00 (9.94)	−0.34
PRI	2024	25	100.2 (8.71)	0.01
PSI	2024	28	88.14 (8.57)	−0.75
VCI	2024	28	98.66 (12.27)	−0.17
VPI	553	3	101.4 (4.02)	0.07
WMI	2024	28	92.29 (8.76)	−0.53
ID	*n*	k	M	SMD
FSIQ	335	7	55.41	−3.34
PRI	335	7	64.53	−2.03
PSI	335	7	62.95	−2.61
VCI	335	7	66.00	−2.85
WMI	335	7	59.98	−2.84
ASD+ID	*n*	k	M	SMD
FSIQ	102	3	53.35 (1.93)	−3.87
PRI	102	3	66.98 (5.33)	−2.86
PSI	102	3	64.71 (1.85)	−2.98
VCI	102	3	61.18 (0.64)	−2.78
WMI	102	3	59.81 (3.39)	−2.92

Notes: *n* = number of participants grouped; k = number of samples collected; M = mean; SD = standard deviation; SMD = Standard mean difference.

**Table 3:** Meta-regression model stratified by diagnosis.

Group	β	SE	Z	*p*	IC
ID (intercept)	67.50	2.29	29.40	5.17 × 10^−190^	63.00, 72.0
ASD **	26.01	2.55	10.20	1.9762 × 10^−24^	21.01, 31.0
ASD+ID ^1^	−4.67	2.66	−1.75	0.07963222	−9.89, 0.55

** *p* < 0.01; ^1^ *p* > 0.05.

**Table 4:** Meta-regression model stratified by test type.

Group	β	SE	Z	*p*	IC
WAIS-III (intercept)	82.88	3.36	24.63	5.410	76.28, 89.47
WAIS-IV ^1^	−1.21	4.33	−0.27	0.780	−9.70, 7.28
WISC-IV ^1^	5.95	3.69	1.61	0.107	−1.28, 13.19
WISC-V ^1^	4.38	3.81	1.14	0.250	−3.08, 11.85

^1^ *p* > 0.05.

**Table 5:** Meta-regression model stratified by diagnosis and Index.

Index	β	SE	Z	*p*	IC_Inf
ASD − FRI (intercept)	0.006	0.12	0.05	0.95647192	−0.22, 0.24
ASD − FSIQ **	−0.315	0.07	−4.36	1.321 × 10^−5^	−0.46, −0.17
ASD − PRI	0.034	0.08	0.44	0.66280273	−0.12, 0.19
ASD − PSI **	−0.718	0.07	−9.86	5.9153 × 10^−23^	−0.86, −0.58
ASD − VCI	−0.082	0.07	−1.13	0.25805923	−0.22, 0.06
ASD − VPI	0.051	0.09	0.60	0.55104041	−0.12, 0.22
ASD − WMI **	−0.452	0.07	−6.24	4.3727 × 10^−10^	−0.59, −0.31
Index	β	SE	Z	*p*	IC_Inf
ASD+ID − FSIQ (intercept)	−3.341	0.22	−15.40	1.7456 × 10^−53^	−3.77, −2.96
ASD+ID − PRI **	1.307	0.28	4.71	2.4829 × 10^−6^	0.76, 1.85
ASD+ID − PSI	0.700	0.29	2.41	0.01577728	0.13, 1.27
ASD+ID − VCI	0.486	0.29	1.65	0.0987186	−0.09, 1.06
ASD+ID − WMI	0.496	0.29	1.68	0.09221989	−0.081, 1.07
Index	β	SE	Z	*p*	IC_Inf
ID − FSIQ (intercept)	−3.720	0.29	−12.99	1.4744 × 10^−38^	−4.28, −3.15
ID − PRI **	1.149	0.17	6.61	3.8313 × 10^−11^	0.80, 1.49
ID − PSI **	0.989	0.17	5.63	1.7757 × 10^−8^	0.65, 1.33
ID − VCI **	1.080	0.17	6.19	6.1056 × 10^−10^	0.74, 1.42
ID − WMI **	0.965	0.17	5.43	5.5995 × 10^−8^	0.62, 1.31

** *p* < 0.01.

## Data Availability

All analyzed data are available in the review article and the Supplementary Materials. Original unprocessed data is available in the original selected article. If you wish to access the R code or additional data, please contact the researchers.

## Supplementary Materials

The following supporting information can be downloaded at: https://www.mdpi.com/article/10.3390/ejihpe16010012/s1, Supplement S1: STROBE analysis by heatmap; Figure S1: Heatmap bias in STROBE; Table S1: Heterogenity based on tests, index and diagnosis simultaneosly. Supplement S2: Figure S2: FSIQ forest plot organized by found data; Figure S3: VCI forest plot organized by found data; Figure S4: PRI forest plot organized by found data; Figure S5: PSI forest plot organized by found data; Figure S6: WMI forest plot organized by found data; Figure S7: VPI forest plot organized by found data; Figure S8: FRI forest plot organized by found data; Figure S9: Mean in Index and FSIQ based on diagnosis and type of test using Mean in Weschler’s scale. Supplement S3: Table S2: Index of ASD with Egger and Trim-and-fill results; Figure S10: funnel plor of FSIQ in ASD; Figure S11: Funnel plot of PSI results in ASD; Figure S12: Funnel plot of VCI in ASD; Figure S13: Funnel plot of PRI in ASD; Figure S14: Funnel plot of WMI in ASD; Supplement S4: Figure S15: Certainly analises divide by Diagnosis and Index; Table S3: Sensitivity Analisys of FSIQ; Table S4: Sensitivity Analisys of PRI; Table S5: Sensitivity Analisys of PSI; Table S6: Sensitivity Analisys of VCI; Table S7: Sensitivity Analisys of VPI; Table S8: Sensitivity Analisys of WMI; Table S9: Sensitivity Analisys of VPI; Table S10: Certainly separated by diagnosis and index.

## Abbreviations

The following abbreviations are used in this manuscript:

FSIQ	Full Scale IQ
VCI	Vocabulary Comprehension Index
PRI	Perceptual Reasoning Index
PSI	Processing Speed Index
WMI	Working Memory Index
FRI	Fluid Reasoning Index
VPI	Visual Processing Index
ASD	Autism Spectrum Disorder
ID	Intellectual Disability
IDD	Intellectual Development Disorder
ASD+ID	Autism Spectrum disorder comorbid with Intellectual Development Disorder
