# Supplementary material for: Cognitive Profile of Autism and Intellectual Disorder in Wechsler’s Scales: Meta-Analysis
Source: Eur J Investig Health Psychol Educ. 2026 Jan 14;16(1):12. doi: 10.3390/ejihpe16010012 (PMC12839676; doi:10.3390/ejihpe16010012)
Supplement: Supplementary file 1 [file ejihpe-16-00012-s001.zip › Suplement 3 funnel plots.pdf]

Suplementar Material S3 – Funnel plots of ASD

The following presents the results of funnel plots and the Egger and Trim and Fill tests. However, it is important to note that the indices ASD+ID, ID, and VPI and FRI of ASD did not reach a sample size (k) equal to or greater than 10, making these analyses unfeasible. Table S2 presents the Egger and Trim and Fill tests for the indices FSIQ, PRI, PSI, VCI, and WM in the autistic group.

Tabela S2. Indexs of ASD with egger and Trim and fill results

index	Egger Z	Egger p	Trimfill g	Trimfill CI
FSIQ	0.94	0.34900858	-0.336	-0.55, -0.12
PRI	1.60	0.11095273	-0.185	-0.41, 0.04
PSI	-0.26	0.79317604	-0.751	-0.90, -0.60
VCI	1.78	0.0758912	-0.083	-0.35. 0,18
WMI	0.267	0.78965426	-0.473	-0.66, -0.29

The data above indicated that there were no cases in which Egger's test showed statistical significance ($p < 0.05$), suggesting no significant signs of asymmetry. Additionally, the Trim and Fill tests yielded results close to the original g estimates, suggesting greater stability of findings even with the possibility of missing or unpublished studies.

Next, visual and specific analyses will be conducted for each index. Figure S9 shows the funnel plot of FSIQ results in ASD.

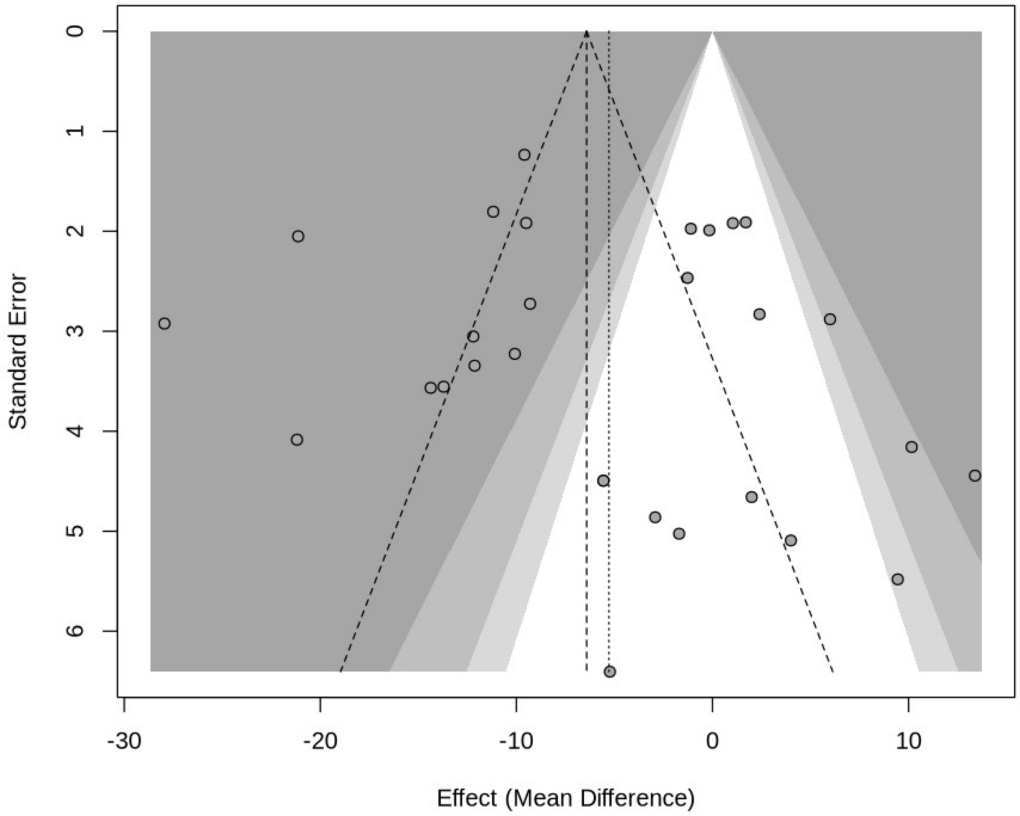

Figure S10. Funnel plot of FSIQ in ASD
Note: Standard error ranges from 0 to 6, while mean difference ranges from -30 to 10. Raw mean differences were used, with 0 marked as a mean of 100. FSIQ = Full-Scale IQ.

Studies were distributed on both sides of the combined effect line, with greater concentration near the central regions and increasing dispersion among less precise estimates. However, there is slight leftward asymmetry, with some studies positioned outside the funnel, especially those with higher standard errors.

To complement the visual inspection, Egger's test was performed, yielding a result (z = 0.94; p = 0.349) that did not indicate statistically significant asymmetry. This suggests that the distribution of effects is compatible with sample variation and heterogeneity among studies, rather than systematic publication bias.

Additionally, the trim-and-fill analysis estimated an adjusted effect of -0.336 (CI: -0.55 to -0.12), close to the original estimate, indicating that the possible absence of studies did not substantially alter the magnitude or direction of the combined effect. Next, Figure S10 presents the data for PSI

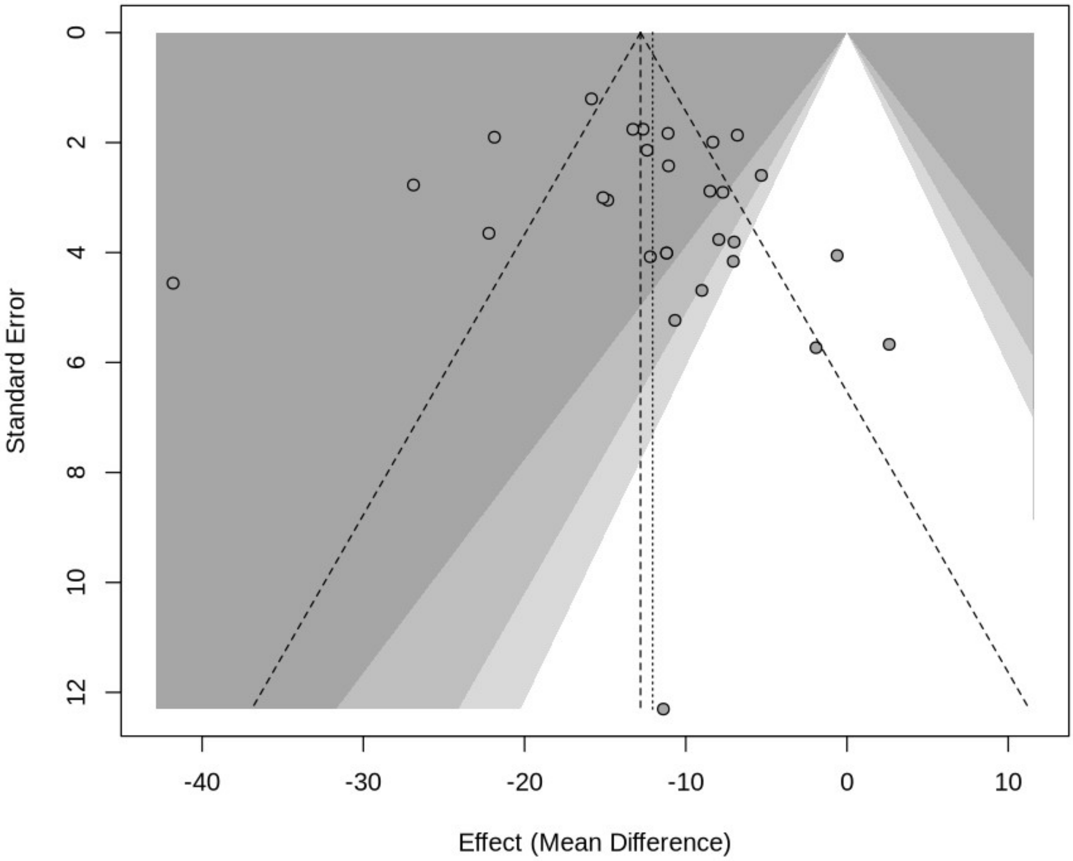

Figure S11. Funnel plot of PSI results in ASD

Visual analysis reveals an asymmetric distribution of studies, with greater concentration on the left side of the graph. There is also dispersion in the lower regions, indicating heterogeneity among individual estimates. However, Egger's test (z = -0.26; p = 0.793) did not show statistically significant asymmetry, suggesting no consistent bias related to study size.

Moreover, the trim-and-fill method estimated an adjusted effect of -0.751 (CI: -0.90 to -0.60), close to original estimates (see article). This typically indicates that the possible absence of studies did not significantly affect the magnitude or direction of the observed effect. Together, visual and statistical analyses suggest that although the distribution shows asymmetry, there is no robust evidence of publication bias that could compromise findings related to PSI in individuals with ASD.

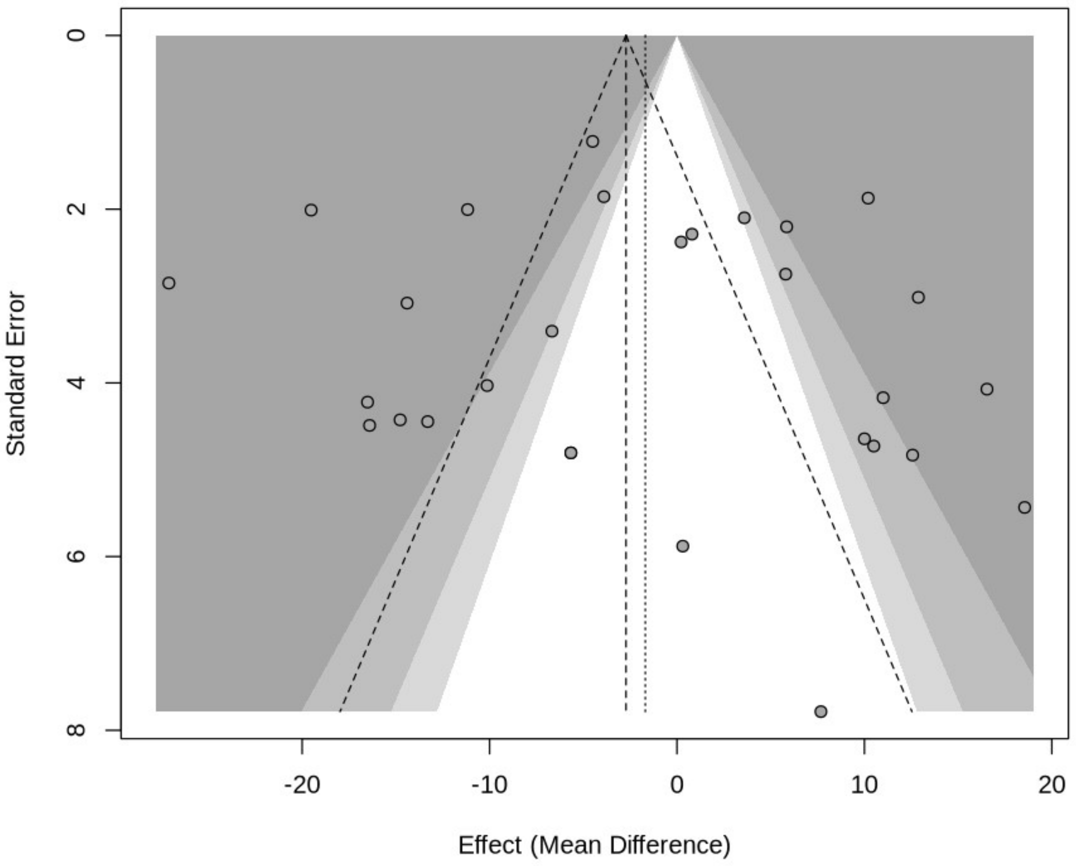

Figure S12. Funnel plot of VCI in ASD

In the funnel plot analysis of VCI, a relatively symmetrical distribution of samples around the combined effect line was observed, although with greater dispersion among less precise studies. Some samples appear shifted to both sides of the funnel margins, suggesting heterogeneity among studies, possibly due to methodological, clinical profile, or sampling differences.

Egger's test yielded a non-significant result ($z = 1.78$; $p = 0.075$), indicating no robust statistical evidence of asymmetry. Thus, the result does not point to bias associated with sample size. Additionally, the trim-and-fill analysis estimated the adjusted effect at -0.083 (CI: -0.35 to 0.18), close to the original estimate and maintaining the direction of the effect. This finding suggests that even with potentially missing studies, the direction of the effect would not undergo intense or significant change.

Therefore, the combination of visual inspection and statistical tests indicates no significant publication bias for the VCI outcome, and the results can be considered consistent within the expected heterogeneity found in the review.

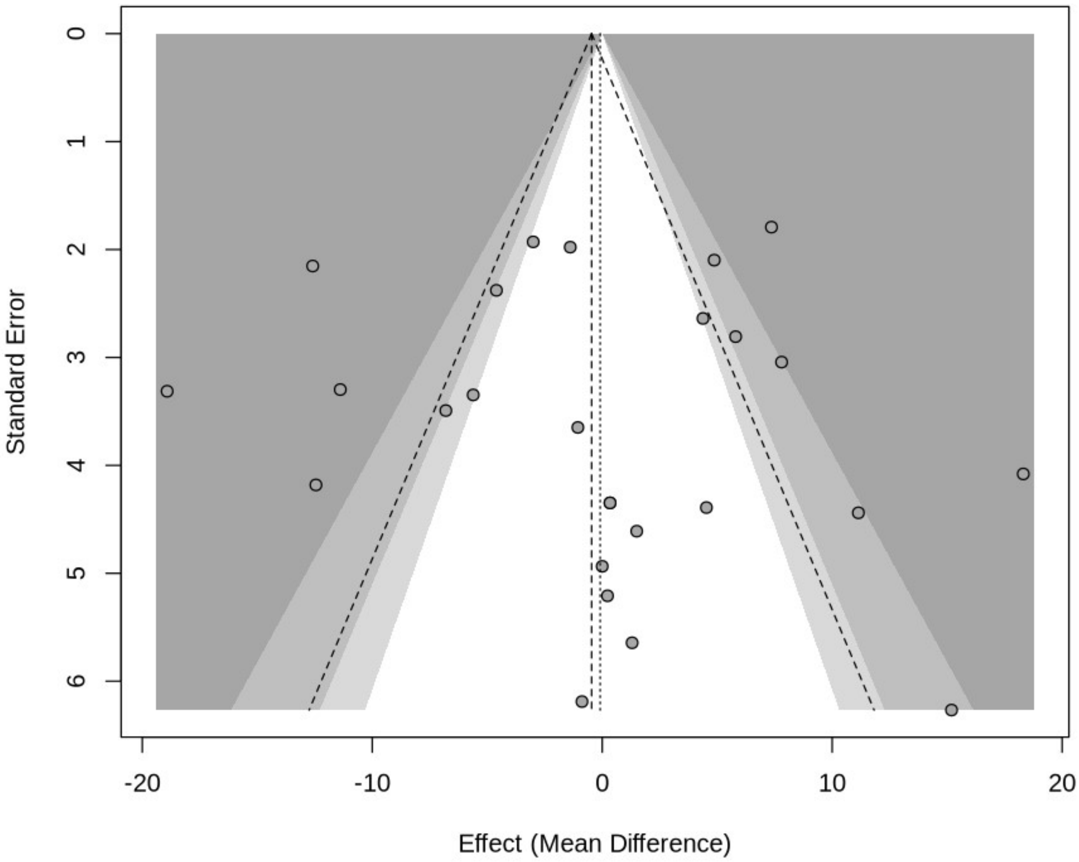

Figure S13. Funnel plot of PRI in ASD

In the case of PRI, distributions appear closer around the line and tend toward both sides of the effect (similar to VCI). Points are more concentrated in the upper axis, with standard error scaling only up to 6. Samples spread across both sides of the central axis, with greater concentration in the upper region. There is some lateral dispersion among less precise studies, suggesting recurrently cited heterogeneity.

Egger's test yielded z = 1.60 (p = 0.110), not indicating statistically significant asymmetry. Although there is a slight leftward shift, the p-value above 0.05 suggests no small-sample bias from a statistical standpoint. The trim-and-fill analysis estimated an adjusted effect of -0.185 (CI: -0.41 to 0.04). The value remains close to the original combined effect, indicating that the hypothetical inclusion of missing studies would have minimal impact on the magnitude of the overall estimate.

Thus, the combination of graphical evaluation and formal tests indicates no robust evidence of publication bias for the PRI outcome in the conducted meta-analysis.

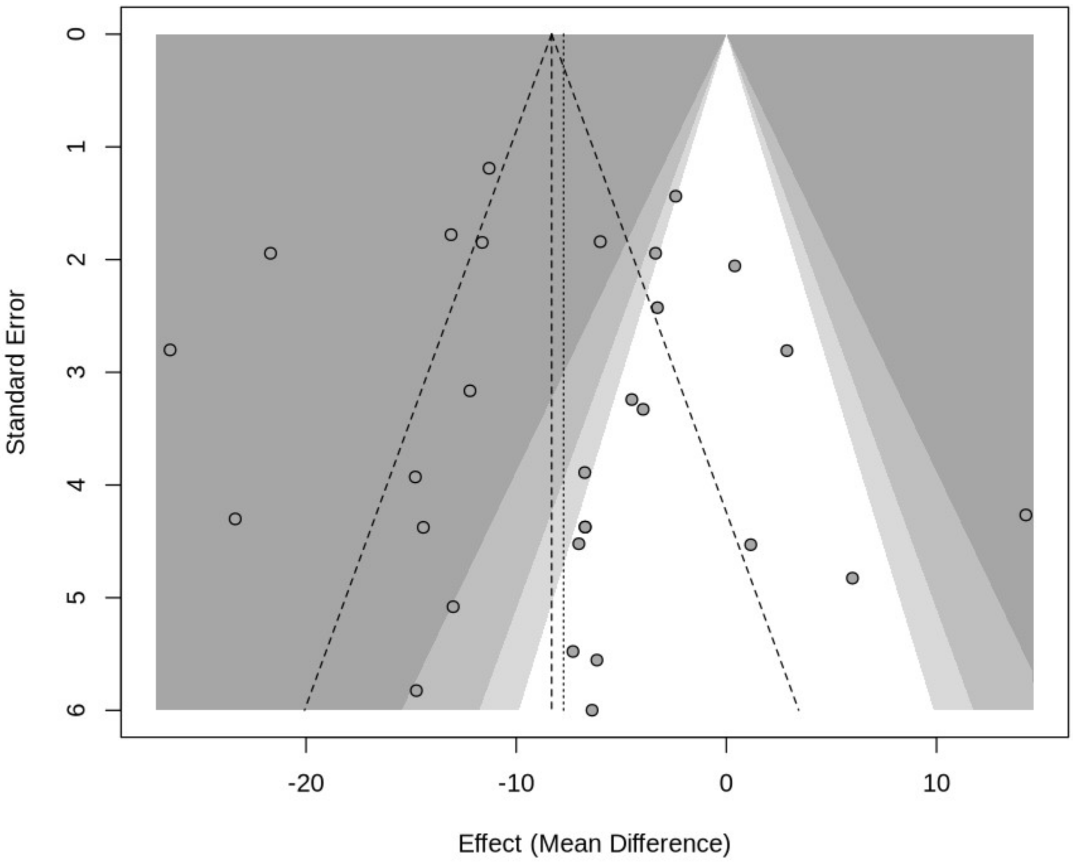

Figure S14. Funnel plot of WMI in ASD

Differently, WMI showed a distribution concentrated to the left of the effect line, with greater dispersion among studies. Additionally, more samples were located at the lower edge (standard error between 4 and 6). Some data also crossed the 0 mark of mean difference.

Egger's test yielded a non-significant result (z = 0.267; p = 0.7897), indicating no statistically consistent asymmetry. This finding reinforces the interpretation that the observed distribution results more from variability among studies than from small-sample bias.

Furthermore, the trim-and-fill analysis estimated an adjusted effect of -0.473 (CI: -0.66 to -0.29), practically equivalent to the originally observed value. This suggests that the eventual inclusion of missing studies would not significantly alter the direction of the effect.
