# Supplementary material for: Cognitive Profile of Autism and Intellectual Disorder in Wechsler’s Scales: Meta-Analysis
Source: Eur J Investig Health Psychol Educ. 2026 Jan 14;16(1):12. doi: 10.3390/ejihpe16010012 (PMC12839676; doi:10.3390/ejihpe16010012)
Supplement: Supplementary file 1 [file ejihpe-16-00012-s001.zip › Suplementar 2 - Indexs.pdf]

Suplementar material S2 – Index and forest-pots

The following plots represent the analyses of each article, across each cognitive index and appropriate diagnosis. Accordingly, all forest plots presented below include the study, diagnosis, test, total number of participants, mean performance, standard deviation, and mean difference (g), along with a 95% confidence interval. An important note is that studies appearing more than once by name represent distinct samples that met the inclusion criteria of the review. A figura S2 apresenta os resultados específicos para Full-scale Intelelctual quociente (FSIQ).

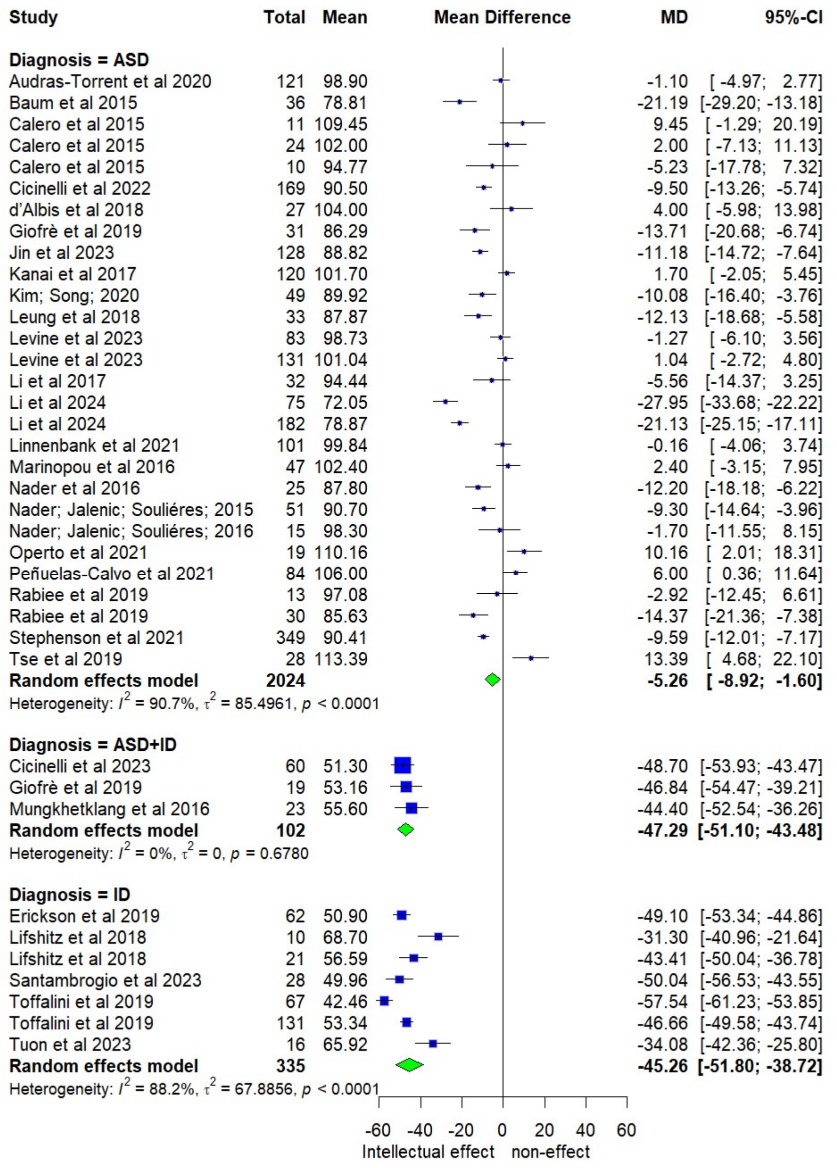

Figura S2. Forest ploto of the mean difference in the FSIQ results considering a expectated control. Note: Random effects model with Hartund-Knapp. ASD = Autism Spectrum Disorder; ID = Intellectual Development Disorder; 95% IC = Confidence Interval with 95%; MD = Mean difference; I² = Heterogenity;

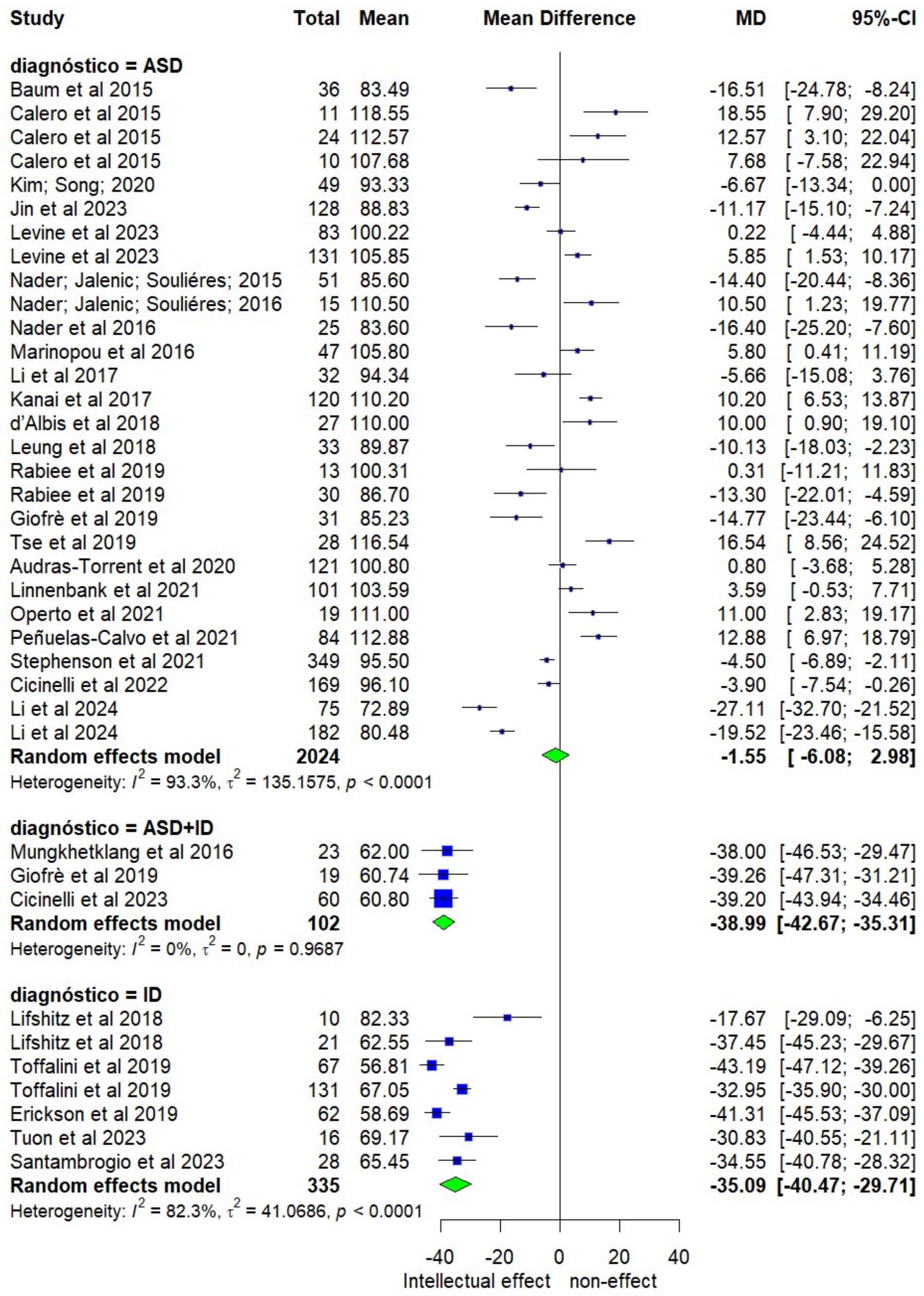

Figrua S3. Forest ploto of the mean difference in the VCI results considering a expectated control

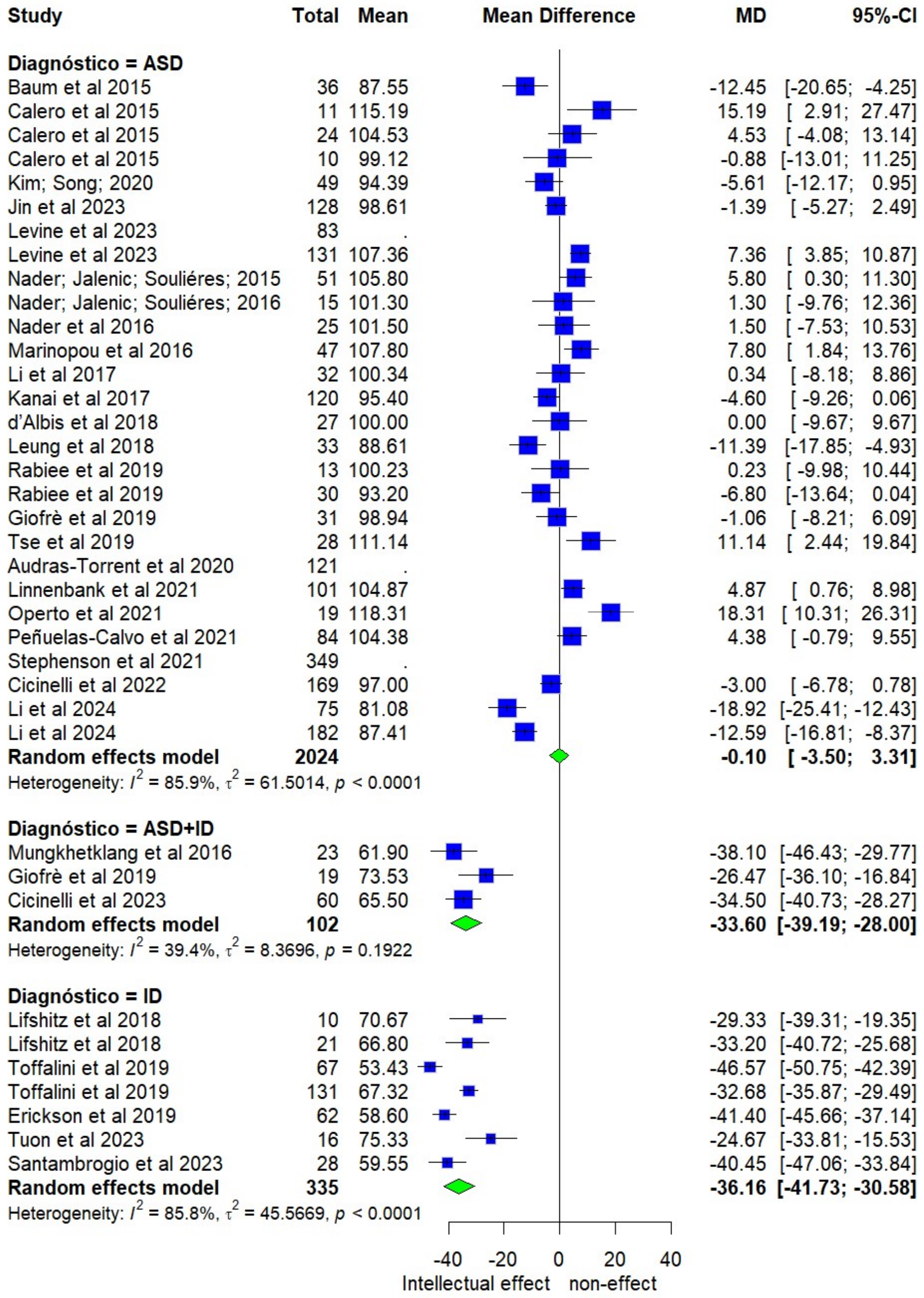

Figura S4. Forest ploto of the mean difference in the PRI results considering a expectated control

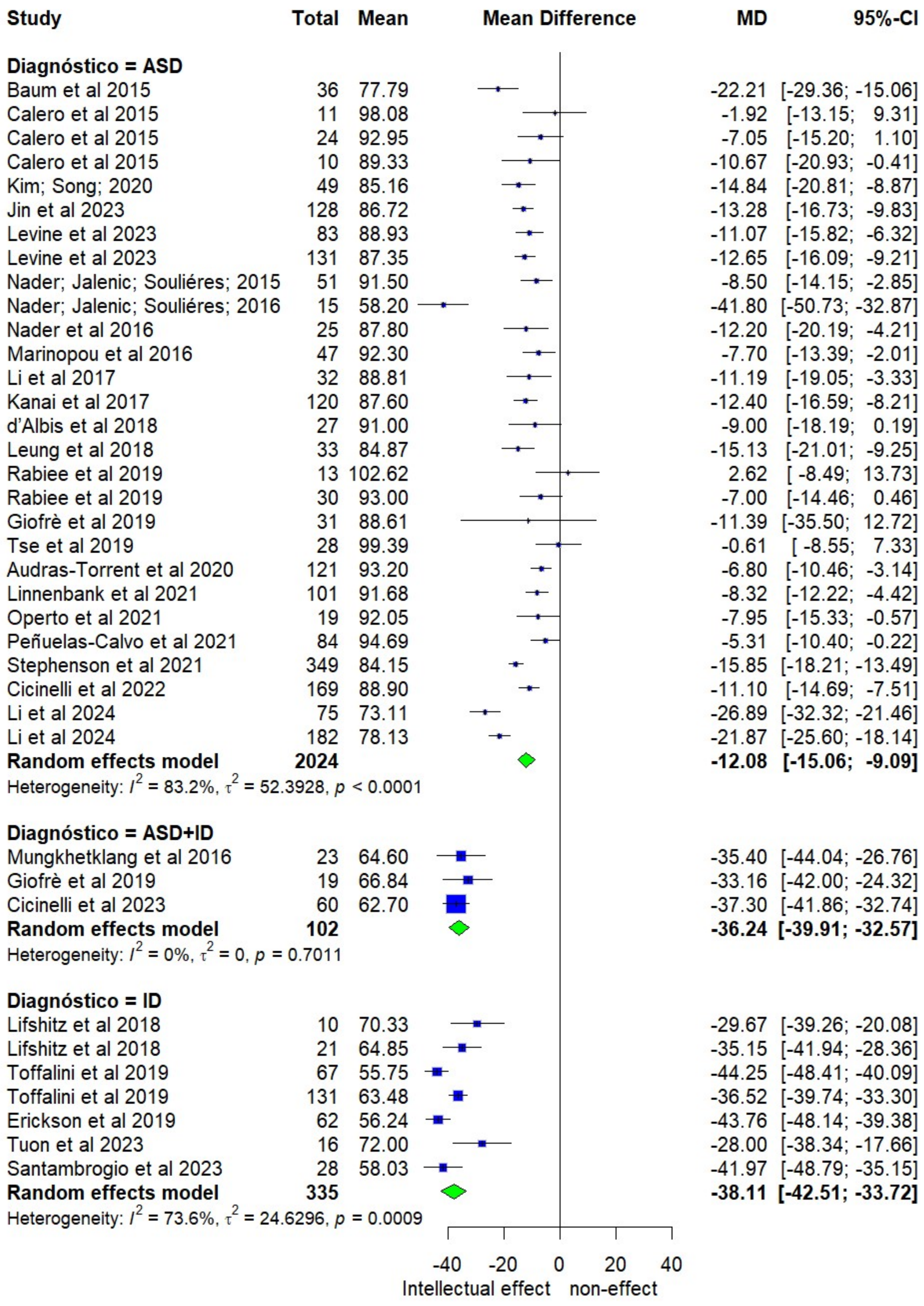

Figura S5. Forest ploto of the mean difference in the PSI results considering a expectated control

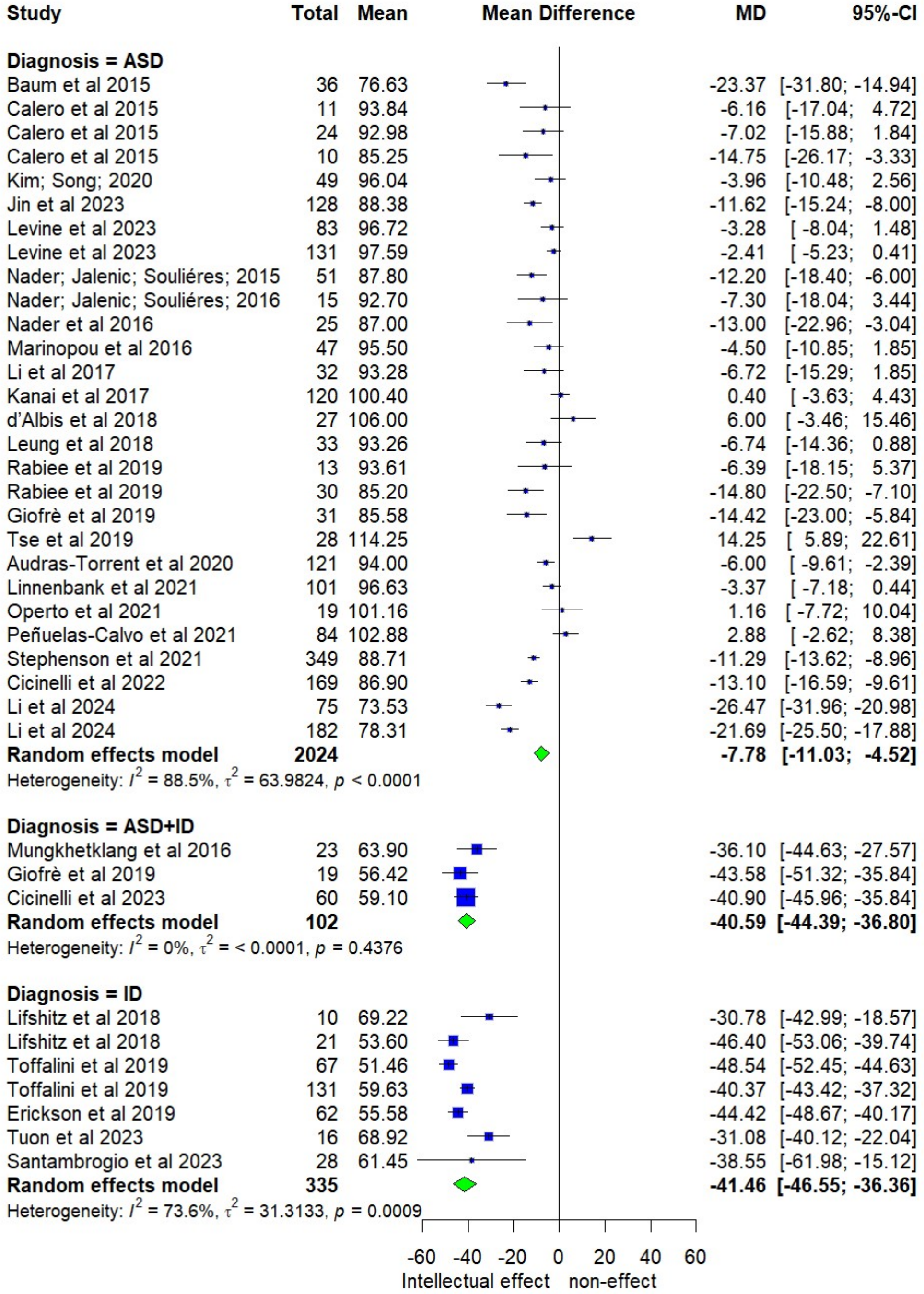

Figura S6. Forest ploto of the mean difference in the WMI results considering a expectated control

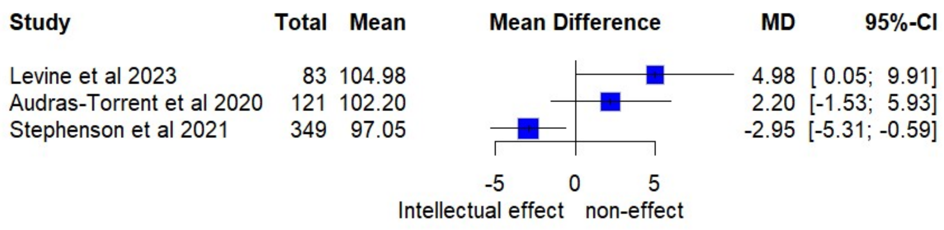

Figura S7. Forest ploto of the mean difference in the *VPI* results considering a expectated control

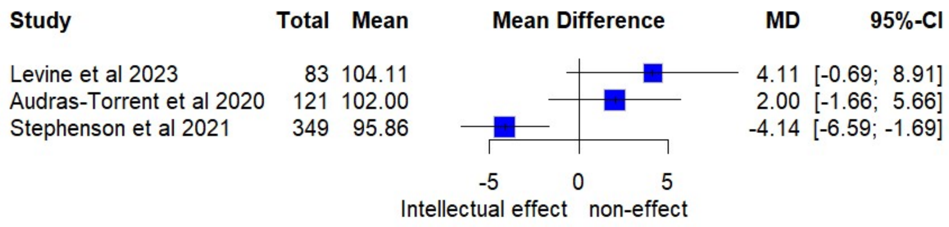

Figura S8. Forest ploto of the mean difference in the *FRI* results considering a expectated control

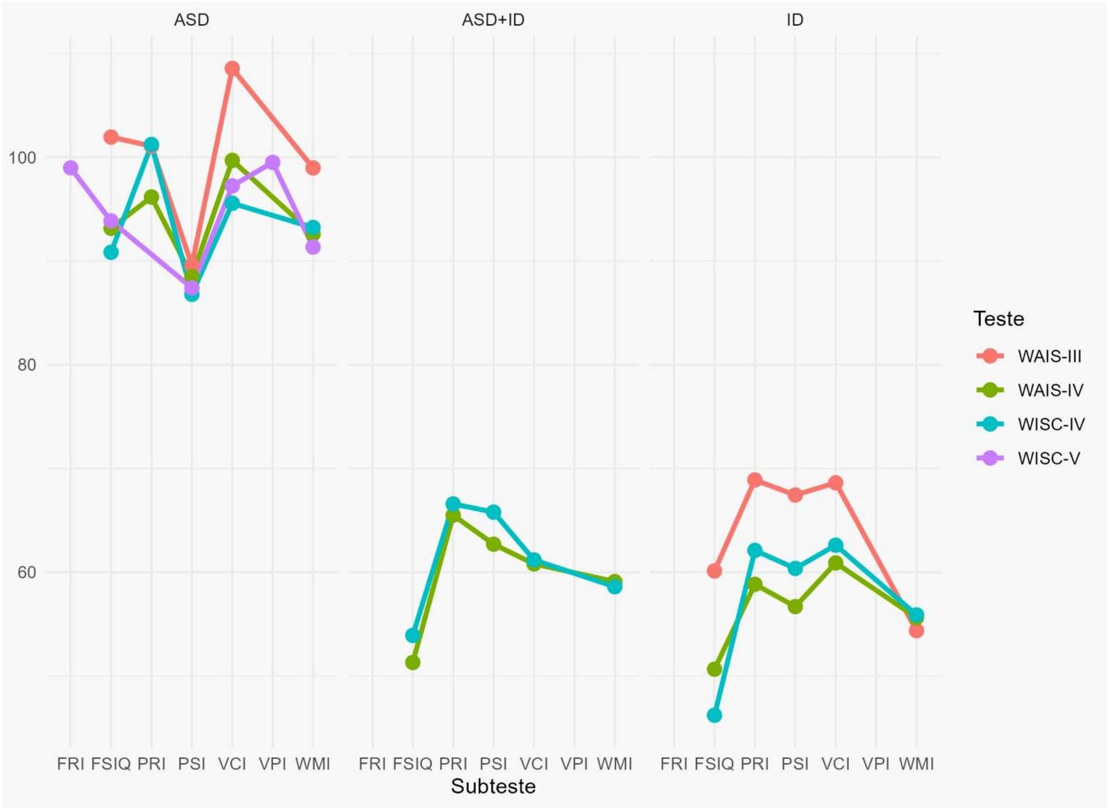

Figura S9. Mean in Index and FSIQ based on diagnosis and type of test using Meand in Weschler's scale
