# Supplementary material for: Cognitive Profile of Autism and Intellectual Disorder in Wechsler’s Scales: Meta-Analysis
Source: Eur J Investig Health Psychol Educ. 2026 Jan 14;16(1):12. doi: 10.3390/ejihpe16010012 (PMC12839676; doi:10.3390/ejihpe16010012)
Supplement: Supplementary file 1 [file ejihpe-16-00012-s001.zip › Suplementar material 4 - Sensitivity and Confiabilidade.pdf]

Suplementar material – bias and Heterogenity

1. *Sensitivity*

Tabel S3. Sensitivity Analisys of FSIQ

Index	Left out Author	year	Test	Diagnosis	k	g_RE	CI_lower	CI_upper	tau2
FSIQ	Levine et al	2023	WISC-V	ASD	38	-1.15385	-1.50165	-0.80604	1.102869
FSIQ	Audras-Torrent et al	2020	WISC-V	ASD	38	-1.15484	-1.5047	-0.80499	1.116729
FSIQ	Stephenson et al	2021	WISC-V	ASD	38	-1.14567	-1.51528	-0.77606	1.256426
FSIQ	Baum et al	2015	WISC-IV	ASD	38	-1.12145	-1.46601	-0.77688	1.081986
FSIQ	Calero et al	2015	WISC-IV	ASD	38	-1.16724	-1.50895	-0.82553	1.065856
FSIQ	Calero et al	2015	WISC-IV	ASD	38	-1.15652	-1.50013	-0.8129	1.076048
FSIQ	Calero et al	2015	WISC-IV	ASD	38	-1.14199	-1.48499	-0.79899	1.074777
FSIQ	Kim; Song;	2020	WISC-IV	ASD	38	-1.13806	-1.48484	-0.79128	1.09643
FSIQ	Jin et al	2023	WISC-IV	ASD	38	-1.13598	-1.48879	-0.78317	1.137214
FSIQ	Levine et al	2023	WISC-IV	ASD	38	-1.15837	-1.50682	-0.80993	1.106999
FSIQ	Nader; Jalenic; Souliéres;	2015	WISC-IV	ASD	38	-1.1369	-1.48381	-0.78998	1.097329
FSIQ	Nader; Jalenic; Souliéres;	2016	WISC-IV	ASD	38	-1.14897	-1.4924	-0.80554	1.076047
FSIQ	Nader et al	2016	WISC-IV	ASD	38	-1.12394	-1.46803	-0.77984	1.079539
FSIQ	Marinopou et al	2016	WAIS-III	ASD	38	-1.15934	-1.50369	-0.81498	1.079931
FSIQ	Li et al	2017	WISC-IV	ASD	38	-1.14601	-1.49116	-0.80087	1.085848
FSIQ	Kanai et al	2017	WAIS-III	ASD	38	-1.15936	-1.50669	-0.81203	1.099419
FSIQ	d'Albis et al	2018	WAIS-IV	ASD	38	-1.1591	-1.50257	-0.81563	1.074808
FSIQ	Leung et al	2018	WAIS-IV	ASD	38	-1.13043	-1.47557	-0.78528	1.085954

FSIQ	Rabiee et al	2019	WISC-IV	ASD	38	-1.14581	-1.48913	-0.80249	1.07583
FSIQ	Rabiee et al	2019	WISC-IV	ASD	38	-1.12638	-1.47102	-0.78174	1.082776
FSIQ	Giofrè et al	2019	WISC-IV	ASD	38	-1.12804	-1.47287	-0.7832	1.084002
FSIQ	Tse et al	2019	WAIS-IV	ASD	38	-1.17375	-1.51451	-0.83299	1.056649
FSIQ	Linnenbank et al	2021	WISC-IV	ASD	38	-1.15597	-1.50414	-0.80781	1.105218
FSIQ	Operto et al	2021	WISC-IV	ASD	38	-1.17189	-1.51322	-0.83057	1.061385
FSIQ	Peñuelas-Calvo et al	2021	WISC-IV	ASD	38	-1.16385	-1.50756	-0.82013	1.075058
FSIQ	Cicinelli et al	2022	WAIS-IV	ASD	38	-1.14361	-1.50007	-0.78715	1.162548
FSIQ	Li et al	2024	WISC-IV	ASD	38	-1.11164	-1.45564	-0.76765	1.07725
FSIQ	Li et al	2024	WISC-IV	ASD	38	-1.12765	-1.48138	-0.77391	1.143521
FSIQ	Mungkhetklang et al	2016	WISC-IV	ASD+ID	38	-1.07433	-1.4127	-0.73597	1.043433
FSIQ	Giofrè et al	2019	WISC-IV	ASD+ID	38	-1.06196	-1.39932	-0.72459	1.038903
FSIQ	Cicinelli et al	2023	WAIS-IV	ASD+ID	38	-1.06113	-1.39192	-0.73034	0.9908
FSIQ	Lifshitz et al	2018	WAIS-III	ID	38	-1.08953	-1.43	-0.74906	1.061187
FSIQ	Lifshitz et al	2018	WAIS-III	ID	38	-1.05937	-1.3962	-0.72255	1.034902
FSIQ	Toffalini et al	2019	WISC-IV	ID	38	-1.01118	-1.32979	-0.69257	0.914441
FSIQ	Toffalini et al	2019	WISC-IV	ID	38	-1.03487	-1.34263	-0.72711	0.84533
FSIQ	Erickson et al	2019	WAIS-IV	ID	38	-1.04126	-1.36715	-0.71537	0.959674
FSIQ	Tuon et al	2023	WISC-IV	ID	38	-1.08427	-1.42425	-0.74429	1.055345
FSIQ	Santambrogio et al	2023	WAIS-IV	ID	38	-1.05333	-1.38834	-0.71833	1.021648
FSIQ	Li et al	2017	WISC-IV	ASD	38	-1.14601	-1.49116	-0.80087	1.085848

Tabel S4. Sensitivity Analisys of PRI

Index	Left out Author	year	Test	Diagnosis	k	G_RE	CI_lower	CI_upper	Tau²
PRI	Baum et al	2015	WISC-IV	ASD	35	-0.66781	-1.01563	-0.31999	1.016951
PRI	Calero et al	2015	WISC-IV	ASD	35	-0.71064	-1.05366	-0.36762	0.990645
PRI	Calero et al	2015	WISC-IV	ASD	35	-0.69579	-1.0415	-0.35007	1.004432
PRI	Calero et al	2015	WISC-IV	ASD	35	-0.68391	-1.0288	-0.33903	1.002207

PRI	Kim; Song;	2020	WISC-IV	ASD	35	-0.67868	-1.02833	-0.32903	1.027975
PRI	Jin et al	2023	WISC-IV	ASD	35	-0.68728	-1.0437	-0.33086	1.070559
PRI	Levine et al	2023	WISC-IV	ASD	35	-0.70414	-1.04977	-0.35852	1.001777
PRI	Nader; Jalenic; Souliéres;	2015	WISC-IV	ASD	35	-0.70041	-1.04676	-0.35406	1.007083
PRI	Nader; Jalenic; Souliéres;	2016	WISC-IV	ASD	35	-0.68879	-1.03421	-0.34337	1.003936
PRI	Nader et al	2016	WISC-IV	ASD	35	-0.69018	-1.0366	-0.34375	1.008755
PRI	Marinopou et al	2016	WAIS-III	ASD	35	-0.70366	-1.04886	-0.35846	1.000006
PRI	Li et al	2017	WISC-IV	ASD	35	-0.68856	-1.03583	-0.34129	1.013565
PRI	Kanai et al	2017	WAIS-III	ASD	35	-0.68239	-1.03908	-0.3257	1.072365
PRI	d'Albis et al	2018	WAIS-IV	ASD	35	-0.68774	-1.03457	-0.3409	1.011186
PRI	Leung et al	2018	WAIS-IV	ASD	35	-0.66348	-1.0106	-0.31636	1.012724
PRI	Rabiee et al	2019	WISC-IV	ASD	35	-0.68666	-1.03193	-0.3414	1.003439
PRI	Rabiee et al	2019	WISC-IV	ASD	35	-0.67353	-1.02106	-0.326	1.015387
PRI	Giofrè et al	2019	WISC-IV	ASD	35	-0.68586	-1.03325	-0.33846	1.014447
PRI	Tse et al	2019	WAIS-IV	ASD	35	-0.70642	-1.05054	-0.36231	0.994157
PRI	Linnenbank et al	2021	WISC-IV	ASD	35	-0.6989	-1.04799	-0.34982	1.023736
PRI	Operto et al	2021	WISC-IV	ASD	35	-0.72485	-1.06546	-0.38425	0.973893
PRI	Peñuelas-Calvo et al	2021	WISC-IV	ASD	35	-0.69669	-1.04605	-0.34733	1.025598
PRI	Cicinelli et al	2022	WAIS-IV	ASD	35	-0.68535	-1.04613	-0.32457	1.098859
PRI	Li et al	2024	WISC-IV	ASD	35	-0.66099	-1.01082	-0.31117	1.028727
PRI	Li et al	2024	WISC-IV	ASD	35	-0.67194	-1.0336	-0.31028	1.104618
PRI	Mungkhetklang et al	2016	WISC-IV	ASD+ID	35	-0.6162	-0.95555	-0.27684	0.966898
PRI	Giofrè et al	2019	WISC-IV	ASD+ID	35	-0.63982	-0.98308	-0.29657	0.990663
PRI	Cicinelli et al	2023	WAIS-IV	ASD+ID	35	-0.62881	-0.96732	-0.29029	0.958684
PRI	Lifshitz et al	2018	WAIS-III	ID	35	-0.62704	-0.96869	-0.28539	0.985112
PRI	Lifshitz et al	2018	WAIS-III	ID	35	-0.61646	-0.9561	-0.27682	0.969068
PRI	Toffalini et al	2019	WISC-IV	ID	35	-0.57606	-0.89422	-0.25791	0.838437
PRI	Toffalini et al	2019	WISC-IV	ID	35	-0.60899	-0.92592	-0.29205	0.829718
PRI	Erickson et al	2019	WAIS-IV	ID	35	-0.58663	-0.9098	-0.26346	0.867497

PRI	Tuon et al	2023	WISC-IV	ID	35	-0.6377	-0.98066	-0.29474	0.989625
PRI	Santambrogio et al	2023	WAIS-IV	ID	35	-0.60085	-0.93664	-0.26506	0.945071
PRI	Li et al	2017	WISC-IV	ASD	35	-0.68856	-1.03583	-0.34129	1.013565

Tabel S5. Sensitivity Analisys of PSI

Index	Left out Author	year	Test	Diagnosis	k	g	CI_lower	CI_upper	Tau²
PSI	Levine et al	2023	WISC-V	ASD	38	-1.26285	-1.51431	-1.01139	0.542233
PSI	Audras-Torrent et al	2020	WISC-V	ASD	38	-1.26982	-1.52039	-1.01925	0.537598
PSI	Stephenson et al	2021	WISC-V	ASD	38	-1.259	-1.52397	-0.99404	0.609827
PSI	Baum et al	2015	WISC-IV	ASD	38	-1.24164	-1.49027	-0.99302	0.529693
PSI	Calero et al	2015	WISC-IV	ASD	38	-1.2707	-1.51739	-1.024	0.522902
PSI	Calero et al	2015	WISC-IV	ASD	38	-1.2661	-1.51414	-1.01805	0.527285
PSI	Calero et al	2015	WISC-IV	ASD	38	-1.25402	-1.50134	-1.0067	0.526583
PSI	Kim; Song;	2020	WISC-IV	ASD	38	-1.25433	-1.50448	-1.00417	0.536435
PSI	Jin et al	2023	WISC-IV	ASD	38	-1.25713	-1.51151	-1.00274	0.556417
PSI	Levine et al	2023	WISC-IV	ASD	38	-1.25875	-1.51322	-1.00428	0.556838
PSI	Nader; Jalenic; Souliéres;	2015	WISC-IV	ASD	38	-1.26536	-1.51485	-1.01586	0.533087
PSI	Nader; Jalenic; Souliéres;	2016	WISC-IV	ASD	38	-1.20688	-1.4503	-0.96346	0.509258
PSI	Nader et al	2016	WISC-IV	ASD	38	-1.25709	-1.50573	-1.00845	0.530211
PSI	Marinopou et al	2016	WAIS-III	ASD	38	-1.26619	-1.51536	-1.01701	0.531632
PSI	Li et al	2017	WISC-IV	ASD	38	-1.26142	-1.51034	-1.01251	0.530994
PSI	Kanai et al	2017	WAIS-III	ASD	38	-1.26251	-1.51588	-1.00914	0.551421
PSI	d’Albis et al	2018	WAIS-IV	ASD	38	-1.26558	-1.51385	-1.01731	0.528129
PSI	Leung et al	2018	WAIS-IV	ASD	38	-1.24692	-1.49585	-0.99798	0.53125
PSI	Rabiee et al	2019	WISC-IV	ASD	38	-1.2787	-1.52457	-1.03283	0.518386
PSI	Rabiee et al	2019	WISC-IV	ASD	38	-1.26707	-1.51533	-1.01882	0.527859
PSI	Giofrè et al	2019	WISC-IV	ASD	38	-1.27324	-1.52052	-1.02597	0.52303
PSI	Tse et al	2019	WAIS-IV	ASD	38	-1.27773	-1.52398	-1.03149	0.518229
PSI	Linnenbank et al	2021	WISC-IV	ASD	38	-1.26648	-1.51787	-1.01509	0.541742

PSI	Operto et al	2021	WISC-IV	ASD	38	-1.26058	-1.50867	-1.0125	0.5281
PSI	Peñuelas-Calvo et al	2021	WISC-IV	ASD	38	-1.27313	-1.52115	-1.02512	0.525386
PSI	Cicinelli et al	2022	WAIS-IV	ASD	38	-1.26568	-1.52032	-1.01103	0.557561
PSI	Li et al	2024	WISC-IV	ASD	38	-1.23692	-1.4859	-0.98794	0.530397
PSI	Li et al	2024	WISC-IV	ASD	38	-1.25	-1.50599	-0.994	0.564319
PSI	Mungkhetklang et al	2016	WISC-IV	ASD+ID	38	-1.21964	-1.46499	-0.97429	0.51571
PSI	Giofrè et al	2019	WISC-IV	ASD+ID	38	-1.22072	-1.46623	-0.97521	0.517081
PSI	Cicinelli et al	2023	WAIS-IV	ASD+ID	38	-1.19744	-1.43504	-0.95984	0.477397
PSI	Lifshitz et al	2018	WAIS-III	ID	38	-1.22162	-1.46685	-0.97639	0.518642
PSI	Lifshitz et al	2018	WAIS-III	ID	38	-1.20512	-1.44814	-0.9621	0.505791
PSI	Toffalini et al	2019	WISC-IV	ID	38	-1.17729	-1.40721	-0.94736	0.442685
PSI	Toffalini et al	2019	WISC-IV	ID	38	-1.19442	-1.42256	-0.96629	0.433338
PSI	Erickson et al	2019	WAIS-IV	ID	38	-1.18092	-1.41294	-0.9489	0.452218
PSI	Tuon et al	2023	WISC-IV	ID	38	-1.23264	-1.47946	-0.98582	0.523392
PSI	Santambrogio et al	2023	WAIS-IV	ID	38	-1.19938	-1.44086	-0.95789	0.497689
PSI	Li et al	2017	WISC-IV	ASD	38	-1.26142	-1.51034	-1.01251	0.530994

Tabel S6. Sensitivity Analisys of VCI

Index	Left out Author	year	Test	Diagnosis	k	g	CI_lower	CI_upper	Tau^2
VCI	Levine et al	2023	WISC-V	ASD	38	-0.74769	-1.08569	-0.40969	1.047252
VCI	Audras-Torrent et al	2020	WISC-V	ASD	38	-0.74889	-1.08896	-0.40882	1.06086
VCI	Stephenson et al	2021	WISC-V	ASD	38	-0.74154	-1.09953	-0.38355	1.183905
VCI	Baum et al	2015	WISC-IV	ASD	38	-0.72183	-1.05645	-0.38721	1.025878
VCI	Calero et al	2015	WISC-IV	ASD	38	-0.77562	-1.10605	-0.44519	1.002308
VCI	Calero et al	2015	WISC-IV	ASD	38	-0.7648	-1.09668	-0.43291	1.008663
VCI	Calero et al	2015	WISC-IV	ASD	38	-0.75371	-1.086	-0.42143	1.013965
VCI	Kim; Song;	2020	WISC-IV	ASD	38	-0.7361	-1.07259	-0.39961	1.037704
VCI	Jin et al	2023	WISC-IV	ASD	38	-0.72839	-1.0701	-0.38668	1.071889
VCI	Levine et al	2023	WISC-IV	ASD	38	-0.7565	-1.09327	-0.41974	1.038755

VCI	Nader; Jalenic; Souliéres;	2015	WISC-IV	ASD	38	-0.72165	-1.05716	-0.38614	1.031274
VCI	Nader; Jalenic; Souliéres;	2016	WISC-IV	ASD	38	-0.7642	-1.09598	-0.43242	1.009325
VCI	Nader et al	2016	WISC-IV	ASD	38	-0.71913	-1.05279	-0.38547	1.02034
VCI	Marinopou et al	2016	WAIS-III	ASD	38	-0.75828	-1.0919	-0.42465	1.018833
VCI	Li et al	2017	WISC-IV	ASD	38	-0.7385	-1.07355	-0.40346	1.028734
VCI	Kanai et al	2017	WAIS-III	ASD	38	-0.76623	-1.09587	-0.43659	0.992044
VCI	d'Albis et al	2018	WAIS-IV	ASD	38	-0.76103	-1.09357	-0.4285	1.012627
VCI	Leung et al	2018	WAIS-IV	ASD	38	-0.72992	-1.06493	-0.39491	1.028528
VCI	Rabiee et al	2019	WISC-IV	ASD	38	-0.74507	-1.07812	-0.41202	1.017885
VCI	Rabiee et al	2019	WISC-IV	ASD	38	-0.72584	-1.06038	-0.3913	1.025646
VCI	Giofrè et al	2019	WISC-IV	ASD	38	-0.72385	-1.05832	-0.38938	1.025126
VCI	Tse et al	2019	WAIS-IV	ASD	38	-0.7736	-1.10378	-0.44341	0.997362
VCI	Linnenbank et al	2021	WISC-IV	ASD	38	-0.75396	-1.09098	-0.41695	1.040565
VCI	Operto et al	2021	WISC-IV	ASD	38	-0.76641	-1.09801	-0.43481	1.007437
VCI	Peñuelas-Calvo et al	2021	WISC-IV	ASD	38	-0.76487	-1.09631	-0.43342	1.004029
VCI	Cicinelli et al	2022	WAIS-IV	ASD	38	-0.74182	-1.08724	-0.3964	1.096894
VCI	Li et al	2024	WISC-IV	ASD	38	-0.70396	-1.0357	-0.37222	1.006224
VCI	Li et al	2024	WISC-IV	ASD	38	-0.71932	-1.06029	-0.37834	1.066816
VCI	Mungkhetklang et al	2016	WISC-IV	ASD+ID	38	-0.68194	-1.01092	-0.35296	0.991376
VCI	Giofrè et al	2019	WISC-IV	ASD+ID	38	-0.67286	-1.00103	-0.34469	0.987633
VCI	Cicinelli et al	2023	WAIS-IV	ASD+ID	38	-0.66627	-0.98703	-0.34551	0.936588
VCI	Lifshitz et al	2018	WAIS-III	ID	38	-0.71351	-1.04564	-0.38138	1.013719
VCI	Lifshitz et al	2018	WAIS-III	ID	38	-0.67557	-1.00389	-0.34726	0.987829
VCI	Toffalini et al	2019	WISC-IV	ID	38	-0.64617	-0.95999	-0.33236	0.893517
VCI	Toffalini et al	2019	WISC-IV	ID	38	-0.66885	-0.97827	-0.35943	0.865082
VCI	Erickson et al	2019	WAIS-IV	ID	38	-0.65366	-0.97062	-0.33669	0.913027
VCI	Tuon et al	2023	WISC-IV	ID	38	-0.69286	-1.02352	-0.3622	1.003155
VCI	Santambrogio et al	2023	WAIS-IV	ID	38	-0.67302	-1.00017	-0.34587	0.979283
VCI	Li et al	2017	WISC-IV	ASD	38	-0.7385	-1.07355	-0.40346	1.028734

Tabel S7. Sensitivity Analisys of VPI

Index	Left out Author	year	Test	Diagnosis	k	g_RE	CI_lower	CI_upper	Tau²
VPI	Levine et al	2023	WISC-V	ASD	2	-0.03467	-0.35966	0.290321	0.044349
VPI	Audras-Torrent et al	2020	WISC-V	ASD	2	0.041393	-0.43816	0.520944	0.105379
VPI	Stephenson et al	2021	WISC-V	ASD	2	0.211953	0.017268	0.406639	0

Tabel S8. Sensitivity Analisys of WMI

Index	Left out Author	year	Test	Diagnosis	k	g_RE	CI_lower	CI_upper	Tau²
WMI	Levine et al	2023	WISC-V	ASD	38	-1.07639	-1.37108	-0.7817	0.775165
WMI	Audras-Torrent et al	2020	WISC-V	ASD	38	-1.07162	-1.3698	-0.77344	0.79525
WMI	Stephenson et al	2021	WISC-V	ASD	38	-1.06705	-1.37982	-0.75428	0.882577
WMI	Baum et al	2015	WISC-IV	ASD	38	-1.04617	-1.33862	-0.75373	0.76344
WMI	Calero et al	2015	WISC-IV	ASD	38	-1.06566	-1.35689	-0.77443	0.759296
WMI	Calero et al	2015	WISC-IV	ASD	38	-1.06797	-1.36037	-0.77557	0.763656
WMI	Calero et al	2015	WISC-IV	ASD	38	-1.05095	-1.3419	-0.76001	0.758514
WMI	Kim; Song;	2020	WISC-IV	ASD	38	-1.07471	-1.36809	-0.78132	0.768122
WMI	Jin et al	2023	WISC-IV	ASD	38	-1.06156	-1.36104	-0.76208	0.802916
WMI	Levine et al	2023	WISC-IV	ASD	38	-1.07722	-1.37371	-0.78073	0.785364
WMI	Nader; Jalenic; Souliéres;	2015	WISC-IV	ASD	38	-1.06064	-1.35512	-0.76616	0.774465
WMI	Nader; Jalenic; Souliéres;	2016	WISC-IV	ASD	38	-1.06606	-1.35773	-0.7744	0.760734
WMI	Nader et al	2016	WISC-IV	ASD	38	-1.06092	-1.35352	-0.76831	0.764843
WMI	Marinopou et al	2016	WAIS-III	ASD	38	-1.07342	-1.36689	-0.77995	0.768671
WMI	Li et al	2017	WISC-IV	ASD	38	-1.07016	-1.36302	-0.7773	0.765748
WMI	Kanai et al	2017	WAIS-III	ASD	38	-1.08268	-1.37523	-0.79014	0.762572
WMI	d’Albis et al	2018	WAIS-IV	ASD	38	-1.088	-1.37806	-0.79794	0.750029
WMI	Leung et al	2018	WAIS-IV	ASD	38	-1.0691	-1.36211	-0.7761	0.766516
WMI	Rabiee et al	2019	WISC-IV	ASD	38	-1.06736	-1.35878	-0.77593	0.759795

WMI	Rabiee et al	2019	WISC-IV	ASD	38	-1.0545	-1.34729	-0.7617	0.765644
WMI	Giofrè et al	2019	WISC-IV	ASD	38	-1.05814	-1.35115	-0.76512	0.766804
WMI	Tse et al	2019	WAIS-IV	ASD	38	-1.10126	-1.38793	-0.81459	0.730928
WMI	Linnenbank et al	2021	WISC-IV	ASD	38	-1.07583	-1.37157	-0.7801	0.781105
WMI	Operto et al	2021	WISC-IV	ASD	38	-1.08056	-1.3716	-0.78953	0.756346
WMI	Peñuelas-Calvo et al	2021	WISC-IV	ASD	38	-1.08558	-1.37612	-0.79504	0.751297
WMI	Cicinelli et al	2022	WAIS-IV	ASD	38	-1.06185	-1.36386	-0.75983	0.817777
WMI	Li et al	2024	WISC-IV	ASD	38	-1.03827	-1.33029	-0.74624	0.760115
WMI	Li et al	2024	WISC-IV	ASD	38	-1.05059	-1.34999	-0.75119	0.802308
WMI	Mungkhetklang et al	2016	WISC-IV	ASD+ID	38	-1.01799	-1.30642	-0.72956	0.742576
WMI	Giofrè et al	2019	WISC-IV	ASD+ID	38	-0.99784	-1.28367	-0.712	0.730208
WMI	Cicinelli et al	2023	WAIS-IV	ASD+ID	38	-0.99946	-1.28055	-0.71838	0.699517
WMI	Lifshitz et al	2018	WAIS-III	ID	38	-1.0295	-1.31908	-0.73992	0.752142
WMI	Lifshitz et al	2018	WAIS-III	ID	38	-0.98613	-1.27006	-0.70221	0.720062
WMI	Toffalini et al	2019	WISC-IV	ID	38	-0.96368	-1.2334	-0.69396	0.638843
WMI	Toffalini et al	2019	WISC-IV	ID	38	-0.9807	-1.24291	-0.71849	0.598073
WMI	Erickson et al	2019	WAIS-IV	ID	38	-0.97815	-1.25292	-0.70337	0.665619
WMI	Tuon et al	2023	WISC-IV	ID	38	-1.02221	-1.31131	-0.73312	0.747575
WMI	Santambrogio et al	2023	WAIS-IV	ID	38	-1.0574	-1.35017	-0.76463	0.765614
WMI	Li et al	2017	WISC-IV	ASD	38	-1.07016	-1.36302	-0.7773	0.765748

Tabel S9. Sensitivity Analisys of VPI

Index	Left out Author	year	Test	Diagnosis	k	g_RE	CI_lower	CI_upper	Tau²
FRI	Levine et al	2023	WISC-V	ASD	2	-0.07065	-0.45025	0.308951	0.064233
FRI	Audras-Torrent et al	2020	WISC-V	ASD	2	-0.01388	-0.51279	0.485036	0.11521
FRI	Stephenson et al	2021	WISC-V	ASD	2	0.186896	-0.00764	0.381432	0

2. *certainty and Confidence*

Tabel S10. Certainly separated by diagnosis and index

Diagnosis	Index	yi	ci.lb	ci.ub	k	ci_width	certainty
ASD	FSIQ	-0.33	-0.55261909	-0.11673692	29	0.43588217	Low
ASD	VCI	-0.08059221	-0.34260382	0.18141939	29	0.52402321	Low
ASD	WMI	-0.47276627	-0.65662265	-0.2889099	29	0.36771275	Low
ASD	PSI	-0.75108511	-0.90216159	-0.60000864	29	0.30215295	Low
ASD	FRI	0.02705887	-0.28723663	0.34135437	3	0.628591	Low
ASD	VPI	0.06608193	-0.22789869	0.36006256	3	0.58796125	Low
ASD	PRI	0.00926331	-0.18788237	0.20640899	26	0.39429135	Low
ASD+ID	FSIQ	-3.33904986	-3.77151722	-2.90658251	3	0.86493471	Moderate
ASD+ID	VCI	-2.85528775	-3.25139721	-2.45917829	3	0.79221892	Moderate
ASD+ID	PRI	-2.03202869	-2.37459352	-1.68946386	3	0.68512966	Moderate
ASD+ID	WMI	-2.84266663	-3.23897986	-2.44635339	3	0.79262647	Moderate
ASD+ID	PSI	-2.61655724	-3.05414027	-2.17897422	3	0.87516605	Moderate
ID	FSIQ	-3.86803041	-4.47557708	-3.26048374	7	1.21509334	Low
ID	VCI	-2.77837872	-3.33454453	-2.22221292	7	1.1123316	Low
ID	PRI	-2.85822046	-3.36140866	-2.35503227	7	1.00637639	Low
ID	WMI	-2.92202563	-3.84256898	-2.00148228	7	1.8410867	Low

ID	PSI	-2.97993125	-3.42369406	-2.53616844	7	0.88752562	Moderate

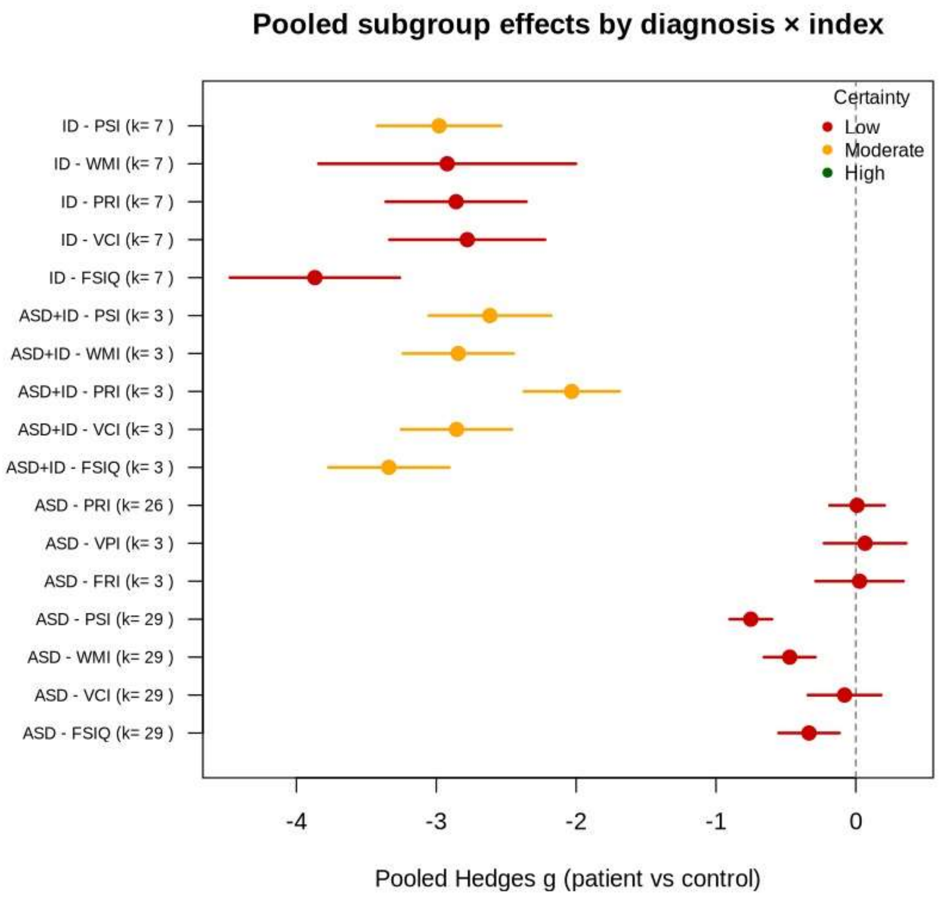

Figure S15. Certainly analises divide by Diagnosis and Index
