# Supplementary material for: Cognitive Profile of Autism and Intellectual Disorder in Wechsler’s Scales: Meta-Analysis
Source: Eur J Investig Health Psychol Educ. 2026 Jan 14;16(1):12. doi: 10.3390/ejihpe16010012 (PMC12839676; doi:10.3390/ejihpe16010012)
Supplement: Supplementary file 1 [file ejihpe-16-00012-s001.zip › Suplementar material bias and hetero.pdf]

Suplementar material – bias and Heterogenity

1. *Bias and Heterogenity addictional data*

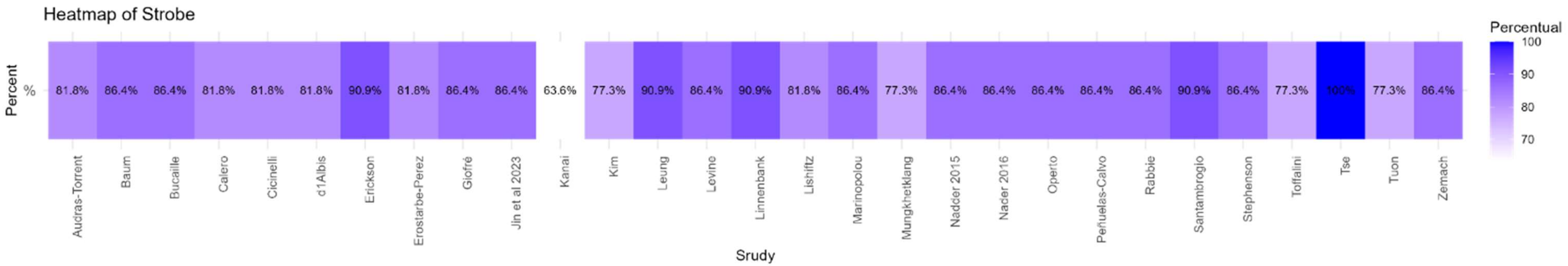

Figura S1. *Heatmap da análise de bias via STROBE*

Suplementary Table S1. Heterogenity based on tests, index and diagnosis simultaneosly

FSIQ	Test	I^2	tau2	Q	p
ASD	ALL	96.24	0.35965	719.4293	0
ASD	WAIS-III	0	0	0.069531	0.79202
ASD	WAIS-IV	95.96	0.502157	74.32515	5.55E-16
ASD	WISC-IV	96.55	0.514557	523.1288	0
ASD	WISC-V	95.11	0.130799	40.91289	1.31E-09
ASD+ID	ALL	14.73	0.005594	2.345729	0.309479
ASD+ID	WAIS-IV	0	0	0	1
ASD+ID	WISC-IV	0	0	0.198557	0.655888
ID	ALL	91.23	0.235015	68.39302	8.73E-13
ID	WAIS-III	83.37	0.320112	6.014959	0.014185
ID	WAIS-IV	0	0	0.001842	0.965769
ID	WISC-IV	95.52	0.368556	44.63067	2.04E-10
**PRI**	**Test**	**I^2**	**tau2**	**Q**	**p**
ASD	ALL	94.39	0.286492	428.0824	0
ASD	WAIS-III	95.67	0.318689	23.1211	1.52E-06
ASD	WAIS-IV	91.59	0.230116	35.68505	8.73E-08
ASD	WISC-IV	95.12	0.357236	368.6899	0
ASD+IDI	ALL	72.47	0.085176	7.264496	0.026457
ASD+ID	WAIS-IV	0	0	0	1
ASD+ID	WISC-IV	84.72	0.246154	6.543431	0.010527
ID	ALL	90.66	0.219442	64.25846	6.11E-12
ID	WAIS-III	0	0	0.913781	0.339113
ID	WAIS-IV	0	0	0.222284	0.637305
ID	WISC-IV	96.036	0.418939	50.45846	1.1E-11
**PSI**	**Test**	**I^2**	**tau2**	**Q**	**p**
ASD	ALL	91.97	0.159887	334.8281	0
ASD	WAIS-III	71.24	0.035688	3.477225	0.062219
ASD	WAIS-IV	81.47	0.092833	16.18578	0.001039
ASD	WISC-IV	93.15	0.249215	262.6487	0
ASD	WISC-V	94.30	0.111275	35.10449	2.38E-08
ASD	ALL	0	0	1.925296	0.38188
ASD	WAIS-IV	0	0	0	1
ASD	WISC-IV	0	0	0.293188	0.588185
ID	ALL	84.42	0.122438	38.50547	8.95E-07
ID	WAIS-III	35.79	0.035577	1.557358	0.212052
ID	WAIS-IV	0	0	0.528937	0.467054
ID	WISC-IV	90.92	0.173082	22.02034	1.65E-05
**WMI**	**Test**	**I^2**	**tau2**	**Q**	**p**
ASD	ALL	95.33	0.286316	578.2412	0
ASD	WAIS-III	72.14	0.0373	3.589123	0.058159
ASD	WAIS-IV	97.17	0.724323	105.881	0
ASD	WISC-IV	95.20	0.363905	375.2374	0
ASD	WISC-V	92.13	0.078696	25.41217	3.03E-06
ASD+ID	ALL	33.98	0.016658	3.02956	0.219857
ASD+ID	WAIS-IV	0	0	0	1
ASD+ID	WISC-IV	57.83	0.060887	2.371197	0.123592

ID	ALL	84.81	0.126147	39.49013	5.74E-07
ID	WAIS-III	89.44	0.540894	9.473789	0.002084
ID	WAIS-IV	72.84	0.066355	3.682491	0.054987
ID	WISC-IV	92.07	0.200817	25.22841	3.32E-06
**VPI**	**Test**	**I^2**	**tau2**	**Q**	**p**
ASD	WISC-V	91.77	0.07493	24.29171	5.31E-06
**FRI**	**Test**	**I^2**	**tau2**	**Q**	**p**
ASD	WISC-V	93.12	0.090992	29.07025	4.87E-07

2. Search Startegy

PsyNet, Science Direct, PubMed, Embase

(ID OR intellectual disability* OR intellectual defficient* OR autism∗ OR ASD OR pervasive development∗ or Asperger∗ AND (WAIS or WISC)

Observational Study Quality Evaluation

PsyNet

Title: ID *OR* **Title**: intellectual disability *OR* **Title**: intellectual defficient *OR* **Title**: autism *OR* **Title**: ASD *OR* **Title**: pervasive development *OR* **Title**: Asperger *AND* **Any Field**: WAIS *OR* **Any Field**: WISC *AND* **Year**: 2015 *To* 2025

Science Direct

(ID OR intellectual disability OR intellectual defficient OR autism OR ASD OR pervasive development or Asperger AND (WAIS or WISC)

Title, abstract ou Keywords: (ID OR intellectual disability OR intellectual defficient OR autism OR ASD OR pervasive development or Asperger

PubMed

ID OR intellectual disability* OR intellectual defficient* OR autism∗ OR ASD OR pervasive development∗ or Asperger∗ AND (WAIS or WISC)

EMBASE

(id:ti OR 'intellectual disability':ti OR (intellectual AND disability) OR 'intellectual defficient':ti OR (intellectual AND defficient) OR autism:ti OR asd:ti OR 'pervasive development':ti OR asperger:ti) AND (wais OR wisc) AND [2015-2025]/py
